# Heat Stress-Dependent Association of Membrane Trafficking Proteins With mRNPs Is Selective

**DOI:** 10.3389/fpls.2021.670499

**Published:** 2021-06-24

**Authors:** Heike Wolff, Marc Jakoby, Lisa Stephan, Eva Koebke, Martin Hülskamp

**Affiliations:** ^1^Cluster of Excellence on Plant Sciences (CEPLAS), Botanical Institute, Cologne University, Cologne, Germany; ^2^Botanical Institute, Biocenter, Cologne University, Cologne, Germany

**Keywords:** SKD1, endosomal trafficking, RNA metabolism, membrane trafficking, ESCRT, heat stress, mRNP

## Abstract

The Arabidopsis AAA ATPase SKD1 is essential for ESCRT-dependent endosomal sorting by mediating the disassembly of the ESCRTIII complex in an ATP-dependent manner. In this study, we show that SKD1 localizes to messenger ribonucleoprotein complexes upon heat stress. Consistent with this, the interactome of SKD1 revealed differential interactions under normal and stress conditions and included membrane transport proteins as well as proteins associated with RNA metabolism. Localization studies with selected interactome proteins revealed that not only RNA associated proteins but also several ESCRTIII and membrane trafficking proteins were recruited to messenger ribonucleoprotein granules after heat stress.

## Introduction

Ubiquitinylated membrane proteins are targeted for degradation in lysosomes by the multivesicular body (MVB) pathway. MVB sorting is governed by proteins of the endosomal complex required for transport (ESCRT). Four complexes, ESCRT0, ESCRTI, ESCRTII, and ESCRTIII, assemble sequentially on endosomes to mediate invagination and fission of the MVBs' outer membrane (Huotari and Helenius, 2011; Gao et al., 2017). This process leads to the formation of intraluminal vesicles (ILVs), whose content is degraded after fusion of the MVB with the lysosome. ESCRTIII is essential for the invagination and fission of intraluminal vesicles. This step involves the ATPase SUPPRESSOR OF K(+) TRANSPORT GROWTH DEFECT 1 (SKD1) and its regulator LYST INTERACTING PROTEIN 5 (LIP5) (Hurley and Hanson, 2010). SKD1 is an ATPase of the AAA (ATPases Associated with diverse cellular Activities) class I. It contains one central ATPase cassette built up by one large, highly conserved AAA-ATPase domain and a smaller, less conserved AAA-ATPase domain (Scott et al., 2005b; Azmi et al., 2006; Vajjhala et al., 2006; Xiao et al., 2007). Endosomal recruitment and the majority of protein interactions occur *via* an N-terminal MIT-domain connected to the ATPase cassette by a long, flexible linker (Babst et al., 1998; Lottridge et al., 2006; Obita et al., 2007; Stuchell-Brereton et al., 2007). When inactive (ADP bound or absence of nucleotides), SKD1 is present in the cytosol as a monomer or homodimer. Recruitment to the endosomal membrane by ESCRTIII core and associated proteins and ATP binding initiates the oligomerization of SKD1 in a barrel-shaped, double-ring structure consisting of 12 or 14 subunits (Hartmann et al., 2008; Landsberg et al., 2009). The activated SKD1 oligomer disassembles the ESCRTIII complex by sequential interactions *via* its MIT-domain. Conformational changes, driven by ATP hydrolysis, detach the proteins from each other and the membrane and release them into the cytosol in their “closed,” monomeric conformation (Scott et al., 2005a). Transient or inducible expression of dominant-negative versions of SKD1 in Arabidopsis results in the formation of aberrantly large MVBs with reduced ILV numbers and reduced transport of vacuolar cargo. Furthermore, the expression of mutated SKD1 under the control of the epidermal cell-specific promoter of GLABRA 2 (GL2) revealed that the loss of SKD1 causes cell expansion phenotypes, vacuolar fragmentation, and inhibits seed coat mucilage production (Shahriari et al., 2010a,b), suggesting that SKD1 null mutants are lethal.

Another positive regulator of SKD1, the Beige and Chediak Higashi (BEACH) domain protein SPI, was found to directly interact with SKD1 and LIP5 and function in membrane trafficking (Saedler et al., 2009; Steffens et al., 2017). In Arabidopsis, it was shown that the SPI protein is not only associated with ESCRT-dependent pathways but also involved in the recruitment of salt-stress responsive mRNAs to processing bodies (P-bodies) and their selective stabilization (Steffens et al., 2015). This indicates that SPI has a dual role in membrane trafficking and RNA metabolism. Recently, the analysis of SPI in *Arabis alpina* has provided evidence that this dual role might be evolutionarily conserved (Stephan et al., 2021). These findings raise the question, whether other ESCRT proteins, or endosomal sorting in general, are associated with mRNA pathways.

Cellular stresses, such as salt, starvation, hypoxia, or heat, trigger polysome disassembly. From there, mRNAs are either transported to P-bodies, or to another, closely related class of messenger ribonucleoprotein (mRNP) granules, the stress granules (SGs) (Anderson and Kedersha, 2008). Both classes of mRNP granules share some associated proteins and have been shown to exchange protein and mRNA content *via* docking and fusion events in mammals and yeast (Kedersha et al., 2005; Buchan and Parker, 2009). Though they are considered somewhat similar, P-bodies are distinguishable from SGs by the presence of DECAPPING PROTEINs (DCPs), which initiate 5′-3′ mRNA decay (Xu et al., 2006; Xu and Chua, 2009). Therefore, P-bodies are thought to primarily function in transcript degradation, while SGs provide a transient cytosolic storage compartment for mRNAs (Buchan and Parker, 2009). However, transport into P-bodies does not always lead to mRNA degradation, since transcripts can re-enter translation after stress removal (Brengues et al., 2005; Bhattacharyya et al., 2006; Aizer et al., 2014). Yeast and mammalian SGs have been distinguished from P-bodies by the presence of the 40S small ribosomal subunit, proteins of the stalled translation initiation complex, as well as Poly(A)-binding proteins (PABs; Kedersha et al., 2005; Anderson and Kedersha, 2008). PABs bind to poly(A) tails of mRNAs and regulate mRNA stability and protein translation (Tian et al., 1991; Kedersha et al., 1999). In *A. thaliana*, PAB2 and PAB8 have been shown to localize to SGs during hypoxia or after heat stress treatment (Weber et al., 2008; Sorenson and Bailey-Serres, 2014; Bhasin and Hülskamp, 2017), and three UBP1 proteins have been shown to reversibly localize in PAB2-containing cytosolic granules during hypoxia (UBP1c and UBP1a) (Sorenson and Bailey-Serres, 2014) and heat stress (UBP1b) (Weber et al., 2008; McCue et al., 2012; Nguyen et al., 2016).

In this study, we show that SKD1 localizes to mRNP granules after heat stress treatment. In addition, our analysis of the SKD1 interactome with and without heat stress revealed differential interactions. The interactome included proteins involved in membrane trafficking as well as proteins associated with RNA metabolism. Most but not all membrane trafficking proteins identified in the interactome showed a similar localization behavior as SKD1, such that they were recruited to stress granules after heat stress treatment. This indicates that the stress-dependent association of membrane trafficking proteins to mRNPs is selective.

## Materials and Methods

### Constructs and Accession Numbers

The plasmid expressing SKD1 (AT2G27600) under its endogenous promoter (SKD1-YFP) was generated by removal of the 35S Cauliflower Mosaic Virus (CaMV) promoter (restriction digestion with AscI/XhoI) from the pEXSG-YFP plasmid (Feys et al., 2005) and the ligation of a fragment containing the sequence of the SKD1 gene (1.2 kb) upstream of the ATG, generated with the primers 5′-GGGGCGCGCCTTGGTTAATTATCACCTAAAATAG-3′ and 5′-CCCTCGAGGGTTTTACAAGAGAAATTGAAATTC-3′.

DCP1-CFP (AT1G08370), mCH-PAB2 (AT4G34110), YFP-UBP1B (AT1G17370), and SKD1-AQ were previously published (Shahriari et al., 2010b; Steffens et al., 2015; Bhasin and Hülskamp, 2017). CFP-UBP1, mCH-SKD1-AQ, SKD1-mCH, DCP5-YFP (AT1G26110), mCH-SNF7.2 (AT2G19830), YFP-SNF7.2, YFP-VPS24.1 (AT5G22950), mCH-VPS24.1, mCH-CHMP1B (AT1G17730), YFP-CHMP1A (AT1G73030), and mCH-CHMP1A were kindly provided by A. Steffens. mCH-UBP1B (AT1G17370) was kindly provided by H. Bhasin.

For the remaining constructs, coding sequences were amplified from Col-0 cDNA to create donor vectors using the Gateway cloning system. Subsequently, LR reactions were carried out to generate YFP-GRF9 (AT2G42590), YFP-FLOT1 (AT5G25250), YFP-UAP56A (AT5G11170), and YFP-NTF2 (AT5G60980) with the destination vector pENSG-YFP (Feys et al., 2005), as well as eIF4B1-YFP (AT3G26400), LOS4-YFP (AT3G53110), CML10-YFP (AT2G41090), SMF-YFP (AT4G30220), SEC13A-YFP (AT3G01340), and ISTL1-YFP (AT1G34220) with the destination vector pEXSG-YFP (Feys et al., 2005).

### Plant Material and Growth Conditions

*A. thaliana* seeds were put on soil or on $\frac{1}{2}$ MS agar plates (Murashige and Skoog, 1962) and stratified for at least 2 d at 4°C in the dark. Afterwards, the plants were grown under long day conditions (16 h light, 8 h darkness) at 21°C and with an average light intensity of 100 ±20 μmol/m^2^s.

All transgenic plants used in this study are in the Col-0 background: 35S::GFP-SKD1 (Haas et al., 2007), 35S::PAB2-mRFP (Sorenson and Bailey-Serres, 2014), 35S::YFP-RHA1 (AT5G45130) (Geldner et al., 2009), 35S::mCH-ARA7 (kindly provided by A. Steffens), and 35S::YFP (kindly provided by I. Schultheiß Araújo). All stable lines co-expressing two markers were obtained by crossing.

The autophagy-deficient mutant *atg5-1* (SAIL_129_B07) has been described before (Thompson et al., 2005). Homozygosity for the T-DNA insertion was confirmed using the T-DNA specific primer SAIL LB1 5′ GCCTTTTCAGAAATGGATAAATAGCCTTGCTTCC-3′ (Sessions et al., 2002) and the gene specific primer 5′-CACCTACATCGAGTGGCAAC-3′. The wild type allele was amplified using the gene specific primer pair 5′-CACCTACATCGAGTGGCAAC-3′ and 5′- CCTTTGTGCAGAACCCGAAA-3′.

### FM4-64 Staining and Heat Treatment

Roots of *A. thaliana* seedlings grown on $\frac{1}{2}$ MS plates (5–7 d) were stained with FM4-64 dye (Synaptored™ C2, Merck) for the identification of membranous structures by confocal microscopy. FM4-64 was added to liquid $\frac{1}{2}$ MS medium to a final concentration of 50 μM. Heat treatment was carried out as previously described (Bhasin and Hülskamp, 2017).

### Monodansylcadaverine (MDC) Staining

*A. thaliana* rosette leaves were stained with MDC (Sigma 30432) to visualize acidic vesicles/autophagosomes by confocal microscopy with a modified protocol from Pu and Bassham (2016). Young leaves were incubated in 0.5 mM MDC in 1xPBS (1l: 8 g NaCl, 0.2 g KCl, 1.44 g Na_2_HPO_4_, 0.24 g KH_2_PO_4_) for 10 min under constant shaking and in darkness. In order to remove excess MDC, the samples were washed twice in 1xPBS for 5 min under constant shaking and in darkness.

### Particle Bombardment and Confocal Microscopy

Expression of plasmids was carried out in rosette leaves of 2 weeks old Arabidopsis seedlings by biolistic transformation (Mathur et al., 2003) and analyzed by confocal laser scanning microscopy after 12–16 h. Confocal laser scanning microscopy was performed with the Leica DM5500 and DM6000 CS Microscopes and documented with the TCS-SPE and TCS-SP8 imaging systems, respectively (Leica Microsystems, Heidelberg, Germany).

### PCC Analysis

Maximum projections of stacks were generated by confocal microscopy. Laser intensities were kept constant within datasets and kept in a range that minimized overexposure but allowed the detection of as many granules as possible. For each combination, 10 cells (transient transformation) or 10 equally sized leaf areas (stable lines) were analyzed. Unlabeled extracellular background signals and labeled cellular structures, which are not of interest in the respective study, can artificially inflate quantified overlaps (Dunn et al., 2011). Therefore, three regions of interest (ROIs) per cell/leaf area were defined, which excluded the majority of membrane and nuclear signals and contained at least five granular structures (if granules were formed) in each channel. For the quantification of the signal overlap, the Pearson's correlation coefficient (PCC) was used which is defined as:

$$PCC=\frac{\sum_{i}\left(A_{i}-\bar{A}\right)\left(B_{i}-\bar{B}\right)}{\sqrt{\sum_{i}{\left(A_{i}-\bar{A}\right)}^{2}\sum_{i}{\left(B_{i}-\bar{B}\right)}^{2}}}$$

where *A*_*i*_ and *B*_*i*_ are signal intensities of pixel *i* in the two compared channels. $\bar{A}$ and $\bar{B}$ refer to mean signal intensities of the whole image for each channel (Manders et al., 1992; Dunn et al., 2011). A PCC of 1 indicates perfect co-occurrence of two signals, a PCC of 0 a random distribution, and a PCC of −1 indicates perfect negative correlation. PCCs for the different ROIs were calculated with the ImageJ Plugin JACoP (Just Another Colocalization Plugin) (Bolte and Cordelières, 2006).

### Co-immunoprecipitation and Mass Spectrometry

Rosette leaves of 2.5 weeks old soil grown plants (not flowering) of the 35S::GFP-SKD1 and the 35S::YFP line were either subjected to heat treatment or kept at RT (control) before harvest (3 replicates each, 12 samples in total). For each sample, leaves of 5 plants were combined. The GFP Isolation kit (Miltenyi Biotec) was used for immunoprecipitation.

To analyze all co-precipitated proteins by liquid chromatography and tandem mass spectrometry analysis (LC-MS/MS analysis), an in-solution/on-bead digest of the proteins was performed. Subsequently, the samples were loaded onto StageTips for removal of salts and other contaminants before LC-MS/MS analysis. The protocols, solutions, chemicals, and styrene-divinylbenzene–reversed phase sulfonate discs-containing C18 StageTips were provided by the Proteomics Core Facility Cologne (http://proteomics.cecad-labs.uni-koeln.de). The LC-MS/MS analysis was performed by the Proteomics Core Facility Cologne using an EASY nLC 1200 UPLC (Thermo Scientific) and a Q-Exactive Plus (Thermo Scientific) mass spectrometer. The mass spectrometry proteomics data have been deposited to the ProteomeXchange Consortium *via* the PRIDE (Perez-Riverol et al., 2019) partner repository with the dataset identifier PXD025028. The raw data of the MS2 spectra was analyzed by S. Müller, Proteomics Core Facility Cologne, using the Maxquant software (version 1.5.2.8.) set to default parameters. As a reference, the Uniprot ARATH.fasta database (download 16.06.2017) was used, which included common contaminants. The protein and peptide spectrum matches (PSM) false discovery rates (FDRs) were estimated using the target-decoy approach (1% Protein FDR and 1% PSM FDR). Only peptides with a length of at least 7 amino acids were counted and the carbamidomethylation of cysteines was included as a fixed modification. Variable modifications (oxidation and acetyl) were included in the analysis. The match between runs option was enabled and used to boost the number of identifications. Label-free quantification was performed using default settings.

### Statistical Analysis

Statistical analysis was done with RStudio (RStudio: Integrated Development for R. RStudio, Inc., Boston, MA). Datasets were tested for normality using Shapiro-Wilk-test. The Welch's two sample *t*-test was used for significance analysis between normally distributed datasets. The Wilcoxon-Mann-Whitney-test was used to compare normally distributed set to not normally distributed sets.

## Results

### SKD1 Changes Its Subcellular Localization After Heat Stress

To analyze a potential link between ESCRTIII proteins and mRNP granules, we initially focused on the question whether the intracellular localization of SKD1 changes upon stress treatments, which are known to induce RNA granule formation.

The SKD1 protein is evenly distributed in the cytosol under normal conditions. Occasionally, it is found in dot-like structures that correspond to MVBs (Haas et al., 2007; Shahriari et al., 2010b). Also, SKD1 was found in the nucleus. It is, however, not clear whether this is an artifact caused by partial degradation when fused with GFP (Haas et al., 2007) or due to a function in nuclear envelope maintenance (Olmos et al., 2015; Vietri et al., 2015).

**Figure 1:**
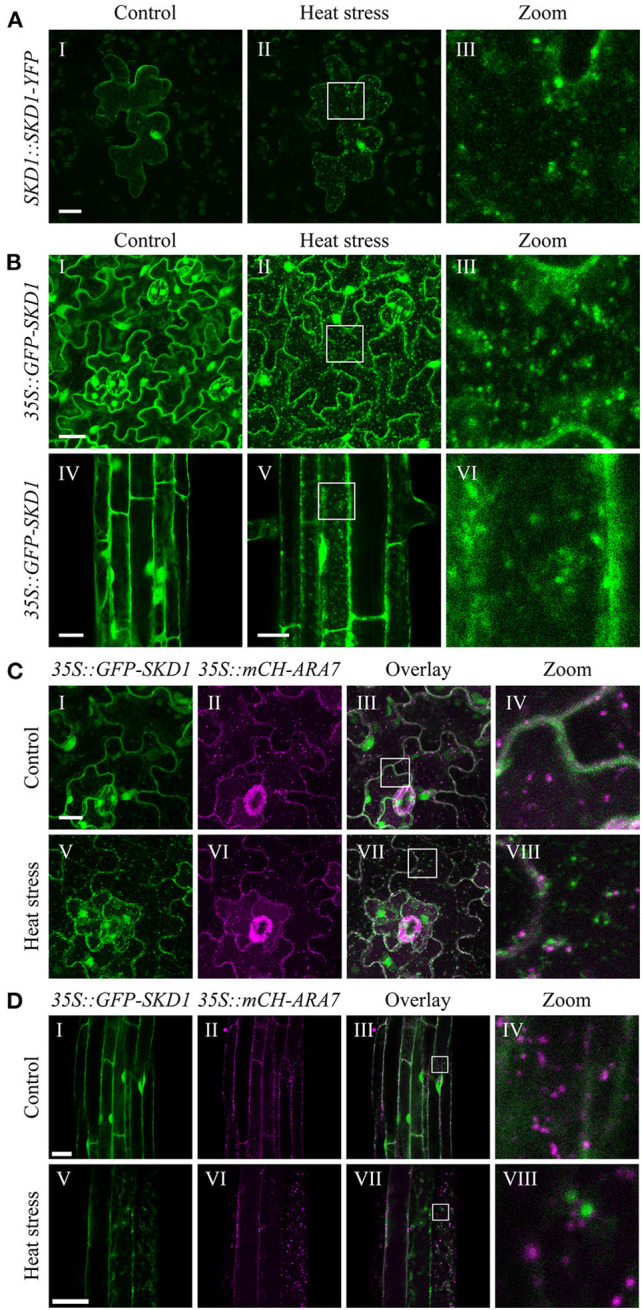
Subcellular localization of SKD1 before and after heat treatment. **(A)** Representative epidermal leaf cell transiently expressing SKD1-YFP under the control of a 1.2 kb upstream sequence of the *AtSKD1* gene (*SKD1::SKD1-YFP*). Images are maximum projections of confocal stacks of the same cell before (I) and immediately after (II) heat stress treatment (40°C, 50 min). Laser intensity was kept constant between images and higher magnifications of the boxes are indicated (III). **(B)** Subcellular localization of GFP-SKD1 in leaf (I–III) and root (IV–VI) epidermal cells of transgenic Col-0 seedlings (*35S::GFP-SKD1*). Plants were grown for 7d on vertically placed $\frac{1}{2}$ MS plates and the same leaves and roots, but not the exact same areas were imaged before and after heat treatment. For leaf cells, maximum projections of stacks are depicted. For root cells, single-plane pictures are shown. Leaf **(C)** and root **(D)** epidermal cells of a transgenic Col-0 line co-expressing GFP-SKD1 and mCH-ARA7 (*35S::GFP-SKD1x35S::mCH-ARA7)* were analyzed for co-localization before (I–IV) and after (V–VIII) heat treatment. Co-localization of signals appears white in the overlay. Scale bar = 20 μm.

To analyze SKD1 localization in *A. thaliana*, we transiently expressed SKD1 fused with YFP at the C-terminus (SKD1-YFP) under the control of a 1.2 kb upstream sequence of the *SKD1* gene in *A. thaliana* leaf epidermal cells by particle bombardment. Consistent with previous reports, we found SKD1-YFP in the cytosol and the nucleus and only occasionally in dot-like structures (Figure 1AI).

To test a possible link of SKD1 to mRNP granules, we analyzed its intracellular localization after a 50 min heat stress treatment at 40°C that leads to mRNP granule formation (Bhasin and Hülskamp, 2017). The overall signal intensity of SKD1-YFP after heat stress was comparable to non-stressed cells, indicating that the stress treatment did not lead to major changes in protein levels. SKD1-YFP signal was found in the cytosol, the nucleus, and cytosolic dot-like structures (Figures 1AII,III). Similar experiments with salt stress or hypoxia did not lead to robust induction of mRNP formation and a distinct localization pattern of the given construct.

We confirmed the heat stress results in a transgenic Col-0 line expressing GFP-SKD1 under the control of the *35S CaMV* promoter (Figure 1B) (Haas et al., 2007). Under non-stress conditions, we found GFP-SKD1 fluorescence in the cytosol and the nucleus (Figures 1BI,IV). After heat stress, GFP-SKD1 was localized in dots in leaf (Figures 1BII,III) and root (Figures 1BV,VI) epidermal cells. Thus, SKD1 protein changes its subcellular localization upon heat treatment in various experimental setups including different transformation method, tissues, expression strength/promoters, and the orientation of modification (C-terminal or N-terminal fusion of the GFP).

### Heat-Induced SKD1 Granules Do Not Co-localize With MVB Markers

The re-localization of SKD1 to small granules under stress conditions could be explained by a more efficient targeting to MVBs. To test this, a stable line was generated that co-expresses GFP-SKD1 and mCHERRY-ARA7/RabF2b (mCH-ARA7) under the *35S CaMV* promoter. ARA7 is a Rab5-related GTPase that serves as a marker for late endosomes (Ueda et al., 2001; Lee et al., 2004). Leaf (Figure 1C) and root (Figure 1D) epidermal cells were analyzed for protein localizations before and after heat treatment. mCH-ARA7 localized in punctate structures in the cytosol with (Figures 1CV–VIII,DV–VIII) and without (Figures 1CI–IV,DI–IV) heat treatment. GFP-SKD1 labeled dots did not co-localize with mCH-ARA7 labeled MVBs after heat stress, indicating that it does not accumulate at late endosomes/MVBs under these conditions. To test whether heat stress causes an association of SKD1 with other membranous structures, roots of the *35S::GFP-SKD1* line were stained with the styryl membrane dye FM4-64 (Synaptored™ C2). After heat stress, we found GFP-SKD1 and FM4-64 labeled dots. These dots were clearly two separate populations with no obvious overlap. A representative staining result (five roots analyzed) is depicted in Supplementary Figure 1. This suggests that the GFP-SKD1 labeled granules are not membrane-coated structures.

### SKD1 Co-localizes With mRNP Granule Marker Proteins After Heat Stress

The finding that GFP-SKD1 does not localize to membranous structures raised the question whether SKD1 might localize to mRNP granules after heat stress. To test this hypothesis, we studied the co-localization of SKD1 with the two P-body markers DCP1 and DCP5 (Xu et al., 2006; Xu and Chua, 2009) and the stress granule markers UBP1B (Lambermon et al., 2000; Weber et al., 2008) and PAB2 (Sorenson and Bailey-Serres, 2014) by transient expression in epidermal cells of Col-0 rosette leaves. SKD1-YFP or SKD1-mCH was co-expressed with DCP1-CFP/DCP5-YFP, mCH-UBP1B, or mCH-PAB2. Unfused YFP was used as a negative control to exclude that the YFP tag mediates the localization behavior (Supplementary Figure 2A).

**Figure 2:**
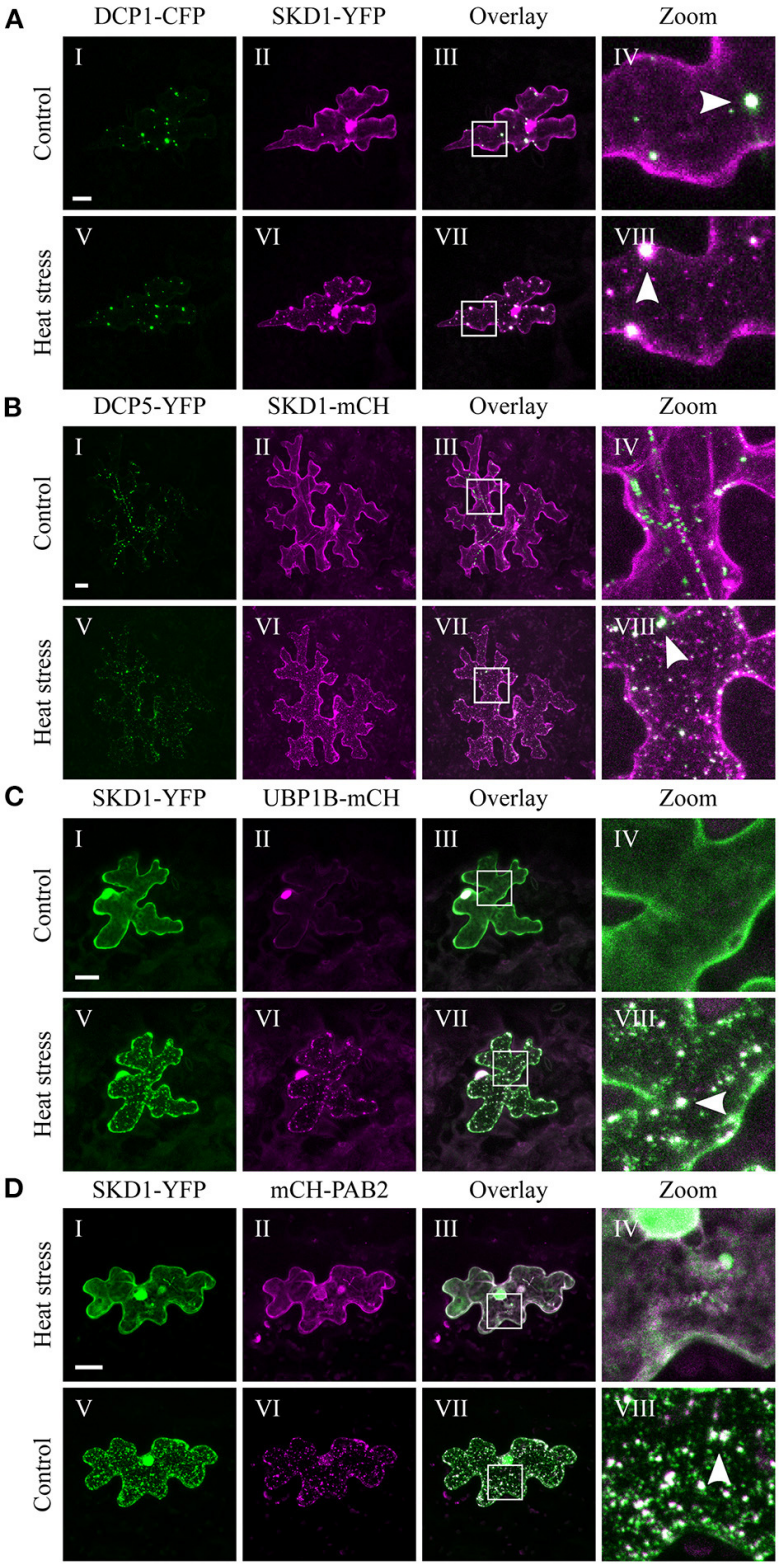
Co-localization of SKD1 with P-body and SG marker proteins. Representative images of transiently transformed epidermal leaf cells that co-express SKD1-YFP or SKD1-mCH with P-body markers **(A)** DCP1-CFP or **(B)** DCP5-YFP, or SG marker UBP1B-mCH **(C)** or mCH-PAB2 **(D)** before (I–IV) and after heat treatment (V–VIII). For clarity, the DCP1-CFP signal is also depicted in green. Arrows indicate co-localizing structures (VIII). Scale bar = 20 μm.

P-bodies are present in the cytosol independent of cellular stress (Xu et al., 2006; Parker and Sheth, 2007). Consistent with this, we found DCP1-CFP (Figure 2A) and DCP5-YFP (Figure 2B) in granular structures before (Figures 2AI,BI) and after heat treatment (Figures 2AV,BV). Occasionally, SKD1 was found in co-localization with DCP1-labeled granules before heat stress. After heat stress, we found substantial co-localization of SKD1-YFP with DCP1-CFP (Figures 2AIV-VIII) and SKD1-mCH with DCP5-YFP (Figures 2BIV-VIII), indicating that stress-induced SKD1 dots overlap with P-bodies.

In contrast to P-bodies, SGs are only seen after the onset of cellular stress (Anderson and Kedersha, 2008). Consistent with the literature, we observed mCH-UBP1B in the nucleus under non-stress conditions and in SGs after heat stress (Lambermon et al., 2000; Weber et al., 2008), and SKD1-YFP clearly co-localized with mCH-UBP1B (Figure 2C). Similar results were obtained with PAB2. The mCH-PAB2 protein localized in the cytosol and granules after heat stress treatment as observed before (Sorenson and Bailey-Serres, 2014; Bhasin and Hülskamp, 2017), and co-localized with SKD1-YFP (Figure 2D).

We confirmed our results using a transgenic *35S::GFP-SKD1* line transiently transformed with mCH-UBP1B (Supplementary Figure 2B). The transformation of single cells by particle bombardment enabled a side-by-side comparison of cells only expressing GFP-SKD1 and cells additionally expressing mCH-UBP1B. GFP-SKD1 strongly co-localized to mCH-UBP1B in the granules after heat stress, and the GFP-SKD1-labeled granules in the neighboring cells resemble the ones in the transiently transformed cell in size and number. This suggests that the co-expression of UBP1B does not influence the localization of GFP-SKD1. In summary, our results indicate that the SKD1 protein associates with mRNP granules during heat stress.

In recent years, several studies addressed the crosstalk between the MVB pathway and the related endomembrane trafficking event macroautophagy (further referred to as autophagy), in which sequestered cargos, desired to be degraded, are delivered to the vacuole *via* double-membrane-bound autophagosomes (Zhuang et al., 2015; Cui et al., 2018). Both pathways highly contribute to and act together in plant stress responses (Wang et al., 2020). In order to exclude potential participation of autophagy in the heat stress-dependent recruitment of SKD1, we investigated stress granule formation and SKD1 localization in the autophagy-defective mutant *autophagy 5-1* (*atg5-1*) (Thompson et al., 2005; Yoshimoto et al., 2009; Havé et al., 2018) and studied the localization patterns of SKD1 and acidic vesicles/autophagosomes, visualized by monodansylcadaverine (MDC) staining in Col-0 leaves (Supplementary Figure 3).

In the *atg5-1* mutant, SKD1 still co-localized to UBP1B-marked SGs under heat stress conditions (Supplementary Figure 3A). Moreover, SKD1 did not co-localize with MDC-stained structures (Supplementary Figure 3B). We thus exclude a connection between heat stress-dependent recruitment of SKD1 to SGs and autophagy.

### Quantitative Analysis of the Co-expression of SKD1 With mRNP Granule Markers After Heat Stress

**Figure 3:**
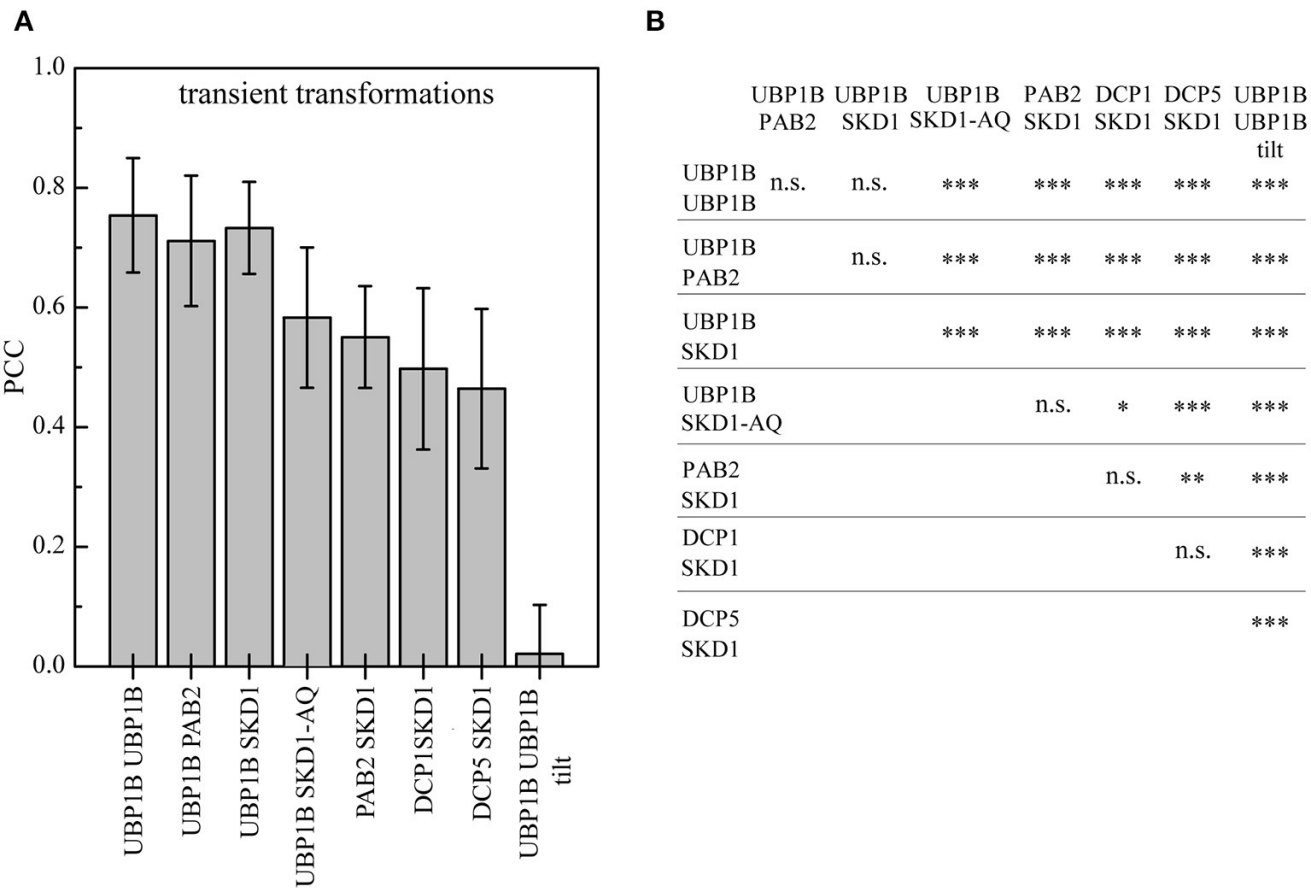
Quantification of SKD1 co-localization with different mRNP granule markers after heat stress. Leaf epidermal cells were transiently double transformed with constructs overexpressing (*35S* promoter) SKD1 in C-terminal fusion with YFP or mCHERRY and an mRNP granule marker (YFP-UBP1B, mCHERRY-PAB2, DCP1-CFP, or DCP5-YFP). The transformed leaves were subjected to heat stress and imaged by CLSM. Maximum projections of stacks were generated. For each combination, three ROIs in 10 cells were analyzed for signal overlap using the Pearson's coefficient (PCC). As positive controls, cells were co-transformed with YFP-UBP1b and mCHERRY-UBP1b or mCHERRY-PAB2. As a negative control, one channel of the YFP/mCHERRY-UBP1b pictures was rotated by 180° before analysis (“tilt”). **(A)** Mean overlaps of the different combinations are depicted in a histogram and error bars indicate standard deviation. **(B)** Statistical significance analysis of co-localizations. Significance levels are indicated as following: n.s., not significant, *p ≤ 0.05, **p ≤ 0.01, ***p ≤ 0.001.

To substantiate the association of SKD1 with different mRNP granule marker proteins, we performed a quantitative analysis by determining the Pearsons's coefficient (PCC, Figure 3) (Manders et al., 1992). To determine the maximal PCC under our conditions (Dunn et al., 2011), we co-expressed YFP-UBP1B with mCH-UBP1B, or YFP-UBP1B with mCH-PAB2 (Supplementary Figures 4A,B). These setups revealed a mean correlation coefficient of 0.75 ± 0.1 standard deviation and 0.71 ± 0.11, respectively. As a negative control, we analyzed a random co-localization by rotating one of the two channels by 180° (Dunn et al., 2011). Here we found a correlation coefficient of 0.02 ± 0.08. The correlation coefficient for the co-expression of SKD1 and UBP1B was in the same range as for the two positive controls (0.73 ± 0.08). The correlation coefficient for the co-localization of SKD1 with PAB2 (0.55 ± 0.09), DCP1 (0.5 ± 0.14) and DCP5 (0.46 ± 0.13) were lower than with UBP1b.

In a second set of experiments, we studied the intracellular localization behavior of the mutant SKD1-AQ protein carrying amino acid exchanges in the ATP binding region Lys178 to Ala (K178A) and Glu234 to Gln (E234Q, Supplementary Figure 4C). These mutations diminish ATP binding and hydrolysis activity of SKD1, which leads to a block of MVB biogenesis and endosomal swelling (Babst et al., 1998; Haas et al., 2007; Shahriari et al., 2010b). Similar mutations of yeast SKD1 were shown to enhance membrane association (Babst et al., 1998). Consistent with this, the Arabidopsis SKD1-AQ protein was observed at a high concentration at enlarged endosomes, indicating that SKD1 ATPase function is essential for its dissociation from membranes (Haas et al., 2007; Shahriari et al., 2010b). We also found SKD1-AQ localized to large aggregates. These showed little co-localization with UBP1B after heat shock treatments. Conversely, the overlap of SKD1-AQ with UBP1B was significantly reduced (0.58 ± 0.12, Figure 3B). This suggests that the two compartments do not overlap and that a stronger association of SKD1-AQ to membranes leads to a reduced localization to mRNP granules.

### Co-expression of the Endosomal Markers SKD1, ARA7, and RHA1 With mRNP Granule Markers

The finding that SKD1 co-localizes with mRNP granules upon heat stress raises the question whether this is SKD1 specific or a general property of endosomal compartments. To analyze this, we studied SKD1, ARA7, and RHA1 co-expression with PAB2 and DCP5 in transgenic plants. RHA1 shares a high amino acid similarity with ARA7 and is also commonly used as a MVB marker (Sohn et al., 2003; Lee et al., 2004).

**Figure 4:**
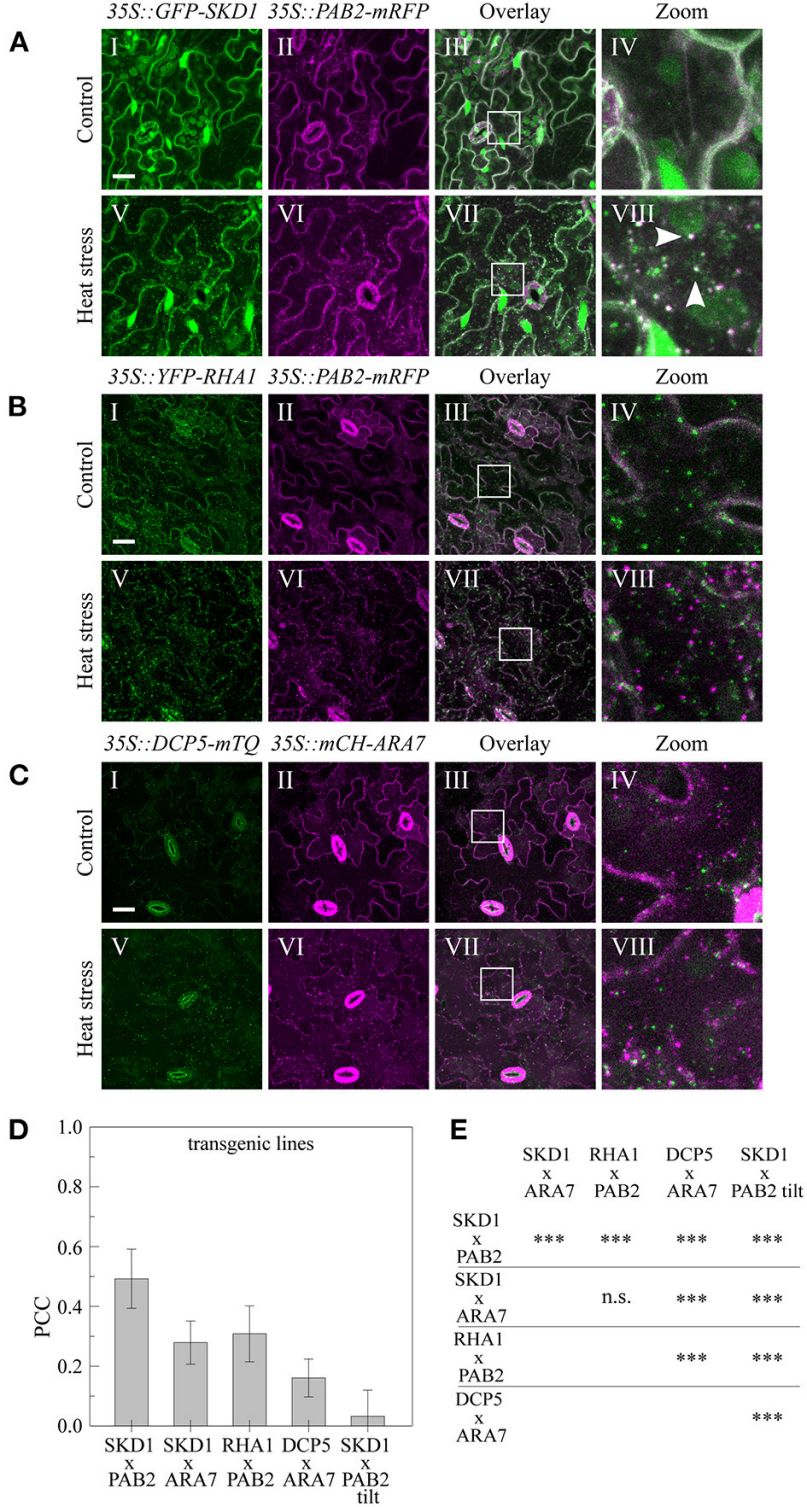
Subcellular localization of endosomal and mRNP granule marker proteins in transgenic lines. Leaves of the generated stable lines **(A)** *35S::GFP-SKD1x35S::PAB2-mRFP*, **(B)** *35S::YFP-RHA1x35S::PAB2-mRFP*, and **(C)** *35S::DCP5-mTQx35S::mCHERRY-ARA7* were imaged by CLSM before (I–IV) and after (V–VIII) heat stress. Scale bar = 20μm. **(D)** Quantification of co-localization in the transgenic marker lines after heat stress. For each line, 10 leaf areas were imaged and PCCs of 3 ROIs per image were measured. As a negative control, one channel of the *35S::GFP-SKD1x35S::PAB2-mRFP* pictures was rotated by 180° before analysis. **(E)** Statistical analysis of co-localization. Significance levels are indicated as following: n.s., not significant, *p ≤ 0.05, **p ≤ 0.01, ***p ≤ 0.001.

The *35S::GFP-SKD1* line crossed to the *35S::PAB2-mRFP* line (Figure 4A) exhibited co-localization of GFP-SKD1 and mRFP-PAB2 after heat shock in a similar range as observed in the transient expression system (PCC: 0.49 ± 0.10, Figure 4D). The analysis of the *35S::GFP-SKD1x35S::mCH-ARA7* (Figure 1C) and *35S::YFP-RHA1x35S::PAB2-mRFP* (Figure 4B) lines revealed significantly lower PCCs (0.28 ± 0.07, 0.31 ± 0.10, Figures 4D,E) than for GFP-SKD1 and PAB2-mRFP. The co-localization coefficients for mCH-ARA7 and DCP5-TURQUOISE (DCP5-mTQ, Figure 4C) was even lower (0.16 ± 0.06). Taken together, our data suggest that the change of SKD1 can be best described with an association with mRNP granules and that endosomal compartments do not generally share this localization behavior.

### Interactome of SKD1

**Figure 5:**
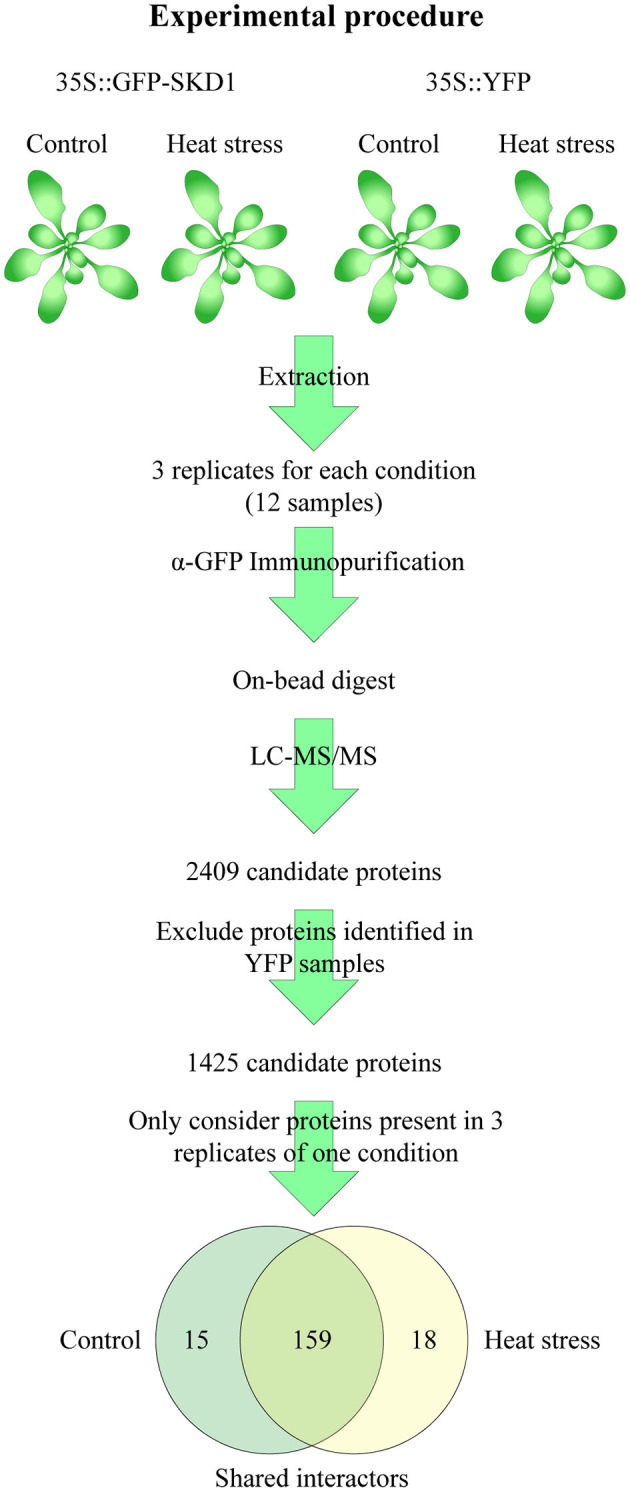
Strategy of the SKD1 interactome identification. Proteins were extracted from rosette leaves of untreated or heat-treated transgenic *35S::GFP-SKD1* and *35S::YFP* plants. The extracted proteins of three replicates of each genotype and condition (total: 12 samples) were subjected to immunoprecipitation using α-GFP-coated magnetic beads. The bound and washed proteins were not eluted but digested on the beads (Lys-C, Trypsin) and subjected to LC-MS/MS analysis. In total, 2,409 proteins were identified. Proteins identified in the YFP replicates were removed from the candidate list, and from the remaining 1,425 candidates, only proteins were considered which were found in three replicates of one condition. This approach resulted in two lists of candidates with a significant overlap. Proteins, which were present in three replicates of one condition and in two replicates of the other, were combined to the SKD1 shared list (159). Proteins, which were not identified or identified only in one replicate in the heat-treated samples, were listed as SKD1 control candidates (15) and vice versa (SKD1 heat stress: 18).

The observation that SKD1 alters its subcellular localization in response to heat stress raises the question whether this is accompanied by a change in protein interactions. To address this, we investigated the heat-dependent *in-vivo* interactome of SKD1 using the transgenic *35S::GFP-SKD1* line. Potential interactors of GFP-SKD1 were co-immunoprecipitated from cell extracts of untreated or heat-treated rosette leaves and analyzed by mass spectrometry (Figure 5). A line overexpressing free YFP (*35S::YFP*) was used as a negative control. For each genotype and condition, proteins of three biological replicates were analyzed. In short, an in-solution digest was performed on the beads, and peptides were subjected to liquid chromatography and tandem mass spectrometry (LC-MS/MS, Dr. S. Müller, CECAD/CMMC Proteomics Facility Cologne). As judged by the highest intensity-based absolute quantification value (iBAQ value, a normalized measurement for protein abundance), SKD1 was the most abundant protein in the *35S::GFP-SKD1* samples. In total, we identified 2,409 proteins (Supplementary Table 1). In a first step, we excluded all proteins that were found in all control samples (*35S::YFP*, control, and heat treatment). In a second step, we selected those proteins from the remaining 1,425 proteins that were found in all three replicates of one condition (Supplementary Table 2).

We distinguished three classes of potential SKD1 interactors. The first class included 15 proteins that were found in all three replicates under normal conditions and in none or only one replicate under heat conditions (called “control,” Figure 5). In the second class, we grouped 18 proteins that were present in all three heat-treated replicates, and none or one of the normal condition replicates (called “heat”). The third class included all 159 proteins that were present in two or three replicates of both conditions (called “shared interactors”). The majority of proteins was identified under both conditions, and no heat-dependent significant differences in protein abundances in the list of shared interactors were identified (*q* ≤ 0.05, Supplementary Table 1). Thus, under our experimental conditions, the SKD1 interactome was moderately changed after heat stress.

For a functional classification of the SKD1 interactors, we did a GO enrichment study of the three classes (SKD1 control, heat, and shared interactors) using the PANTHER classification system (Thomas et al., 2003; Mi et al., 2009). For the SKD1 control set, no overrepresented GO terms (biological process and cellular component) were identified. The SKD1 heat set revealed one overrepresented GO term (biological process: protein folding). In the shared interactor set, we found several overrepresented categories for biological processes and cellular components (Supplementary Figure 5). The enriched GO categories included biological functions related to the known function of SKD1 (intracellular protein transport, vesicle-mediated transport, protein targeting) as well as functional classes with no known relation to SKD1 (e.g., gluconeogenesis, mitochondrial organization, protein folding). A classification of the interactors with respect to cellular compartments revealed mitochondria, the cytosol, and the vacuole as the most enriched categories.

### The SKD1 Interactome Includes RNA-Associated Process and Membrane Trafficking Proteins

The findings that SKD1 is essential for protein trafficking during MVB maturation and that SKD1 is associated with stress granules prompted us to analyze the SKD1 interactome for the presence of potential mRNP granule and membrane trafficking proteins.

Several candidates involved in different RNA-associated processes co-precipitated with SKD1, including several RNA helicases, nucleo-cytoplasmic shuttle proteins, 60S and 30S ribosomal proteins, as well as translation initiation factors (Table 1). Besides, the well-established P-body protein VARICOSE (VCS), a family protein of the *A. thaliana* SG protein CALMODULIN-LIKE 38 (CML38) were found. CML38 is a calcium sensor protein which localizes to SGs during hypoxia in a calcium-dependent manner. In contrast to classical RNA binding proteins, CML38 has no known DNA or RNA binding activity (Jain et al., 2016). We also compared our data set with the interactomes of CML38 and another characterized SG protein in *A. thaliana*, the RNA binding protein RBP45B (Lokdarshi et al., 2016; Muthuramalingam et al., 2017). This revealed several common proteins (Supplementary Table 2). Together, the data supports the idea that SKD1 associates with mRNP granules. Also, we found various co-precipitated membrane trafficking-associated proteins including the ESCRTIII associated components ISTL1 and CHMP1A, vesicle-coating or adaptor proteins, endosomal tethering complex proteins (HOPS and CORVET subunits), vacuolar H^+^-ATPase subunits and proteins, which are transported over an ESCRT-dependent trafficking route or are involved in this process (Aquaporin PIP1 family proteins, PIN polarity establishment proteins, Table 2).

**Table 1:** Proteins of the SKD1 interactome involved in RNA-associated processes.

**Protein**	**AGI code**	**Control/heat** **replicates**	**Annotations and** **descriptions**	**References**	**Shown to be** **in mRNP granules?**
**RNA-associated processes**					
VARICOSE, **VCS**	AT3G13300^a, b^	3/3	P-body component, Decapping complex	TAIR (Xu et al., 2006) (Xu and Chua, 2009)	Yes (*A. thaliana*) (Xu et al., 2006)
UBIQUITIN-SPECIFIC PROTEASE 12, **UBP12**	AT5G06600	3/3	Deubiquitination, JA signaling and circadian clock regulation	TAIR (Ewan et al., 2011)	Related (Mammals) (Xie et al., 2018)
DNA helicase, putative	AT5G67630	3/3	Transcription	TAIR UniProt	Related (Mammals, yeast) (Jain et al., 2016)
LOW EXPRESSION OF OSMOTICALLY RESPONSIVE GENES, **LOS4**	AT3G53110	3/3	DEAD-box ATP-dependent RNA helicase 38, mRNA export from nucleus	TAIR (Gong et al., 2002) (Gong et al., 2005)	No
DEAD-BOX ATP-DEPENDENT RNA HELICASE 56, **UAP56A**	AT5G11170^a^	3/2	RNA helicase, Exon Junction Complex, interacts with mRNA export factors	TAIR (Kammel et al., 2013) (Pfaff et al., 2018)	No
DEK DOMAIN-CONTAINING CHROMATIN ASSSOCIATED PROTEIN, **DEK1**	AT3G48710	3/3	Chromatin remodeling, Exon-Junction Complex	TAIR (Pendle et al., 2005) (Waidmann et al., 2014)	No
EUKARYOTIC INITIATION FACTOR 4A III, **EIF4AIII**	AT3G19760	3/2	DEAD-BOX ATP-DEPENDENT RNA HELICASE 2, Exon Junction Complex, Nonsense-mediated decay, RNA helicase	TAIR (Cui et al., 2016)	No
EUKARYOTIC TRANSLATION INITIATION FACTOR 4B1, **EIF4B1**	AT3G26400	3/0	RNA binding, translation initiation, stimulates activity of eIF4F complex	TAIR (Mayberry et al., 2009)	Yes (Yeast) (Anderson and Kedersha, 2008)
HOMOLOG OF HUMAN KARYOPHENIN SUBUNIT BETA 1, **KPNB1**	AT5G53480	3/2	Putative importin beta, protein import to nucleus	TAIR (Luo et al., 2013)	Yes (Mammals) (Mahboubi et al., 2013)
RAN GTPASE-ACTIVATING PROTEIN 1, **RANGAP1**	AT3G63130	2/3	RAN GTPase activator, involved in nuclear import	TAIR (Jeong et al., 2005) (Rodrigo-Peiris et al., 2011)	No
NUCLEOSOME ASSEMBLY PROTEIN 1;3, **NAP1;3**	AT5G56950	2/3	Nucleosome assembly	TAIR (Dong et al., 2005)	No
60S RIBOSOMAL PROTEIN L10A-3, **RPL10AC**	AT5G22440	3/3	60S ribosomal protein L10A	TAIR	No
30S ribosomal protein, putative	AT5G24490	3/3	30S ribosomal protein, putative	TAIR	No
CALMODULIN-LIKE PROTEIN 10, **CML10**	AT2G41090	0/3	Calmodulin-like calcium-binding protein, involved in ascorbic acid biosynthesis	TAIR (Cho et al., 2016)	Related (*A. thaliana)* (Lokdarshi et al., 2016)
Calmodulin-binding transcription activator 5, **CMTA5**	AT4G16150	3/3	Calcium transcription activator of DREB1 genes	TAIR (Bouché et al., 2002) (Kidokoro et al., 2017)	No
NUCLEAR TRANSPORT FACTOR 2FAMILY PROTEIN, **NTF2**	AT5G60980^b, c^	3/2	(RRM)-containing protein, nucleocytoplasmic transport	TAIR (Parida et al., 2017)	Related
RNA RECOGNITION MOTIF (RRM)-CONTAINING PROTEIN	AT3G23900	0/3	RNA binding	TAIR	Related
SMALL NUCLEAR RIBONUCLEOPROTEIN F, **SMF**	AT4G30220	3/2	Spliceosomal snRNP assembly	TAIR (Kanno et al., 2017)	No

*Protein names, AGI identifiers and number of replicates found under control or heat stress conditions are given. Short descriptions are based on annotations on TAIR, Uniprot or on literature. It is indicated, if proteins or related proteins have been found in mRNP granules in A. thaliana or other species. Furthermore, proteins are labeled, if they have been identified in the interactome of the SG protein CML38 (^a^Lokdarshi et al., 2016), RBP45B (^b^Muthuramalingam et al., 2017), or the ESCRTIII protein VPS2.2 (^c^Ibl et al., 2012). Proteins that were further characterized for their subcellular localization are highlighted in gray*.

**Table 2:** Proteins of the SKD1 interactome involved in membrane trafficking processes.

**Protein**	**AGI code**	**Control/heat** **replicates**	**Annotations and descriptions**	**References**
**Membrane trafficking**				
INCREASED SODIUM TOLERANCE1-LIKE 1, **ISTL1**	AT1G34220	3/2	Regulator of SKD1 activity, ESCRTIII associated	TAIR (Buono et al., 2016)
CHARGED MULTIVESICULAR BODY PROTEIN 1A, **CHMP1A**	AT1G73030^a^	1/3	Regulator of SKD1 activity, ESCRTIII associated	TAIR (Spitzer et al., 2009)
VACUOLAR PROTEIN SORTING 18, **VPS18**	AT1G12470	3/2	CORVET/HOPS complex, endosome to vacuole fusion	TAIR (Takemoto et al., 2018)
VACUOLAR PROTEIN SORTING 41, **VPS41**	AT1G08190	3/2	HOPS complex, endosome to vacuole fusion	TAIR (Hao et al., 2016)
VACUOLELESS1, **VCL1**	AT2G38020	3/3	CORVET/HOPS complex, late endosome to tonoplast transport/fusion	TAIR (Rojo et al., 2001) (Rojo et al., 2003)
TGN-localized SYP41-interacting protein, **TNO1**	AT1G24460	3/3	Vacuolar trafficking/HOPS, putative tethering factor, interacts with SYP41	TAIR (Kim and Bassham, 2011) (Roy and Bassham, 2017)
14-3-3-like protein GF14 omega, **GRF2**	AT1G78300	3/2	Golgi apparatus, plasma membrane, vacuole	TAIR
14-3-3-like protein GF14 mu, **GRF9**	AT2G42590	3/3	PIN polarity establishment	TAIR (Keicher et al., 2017)
ROOTS CURL IN NPA 1, **RCN1**	AT1G25490	3/3	Serine/threonine-protein phosphatase regulatory subunit A alpha isoform, role in PIN polarity by regulation vesicle trafficking of PINs	TAIR (Karampelias et al., 2016)
PLASMA MEMBRANE INTRINSIC PROTEIN 1-1, **PIP1-1**	AT3G61430	3/3	Aquaporin, transmembrane water transporter at PM, ESCRT-dependent trafficking	TAIR (Boursiac et al., 2005) (Wang et al., 2017)
PLASMA MEMBRANE INTRINSIC PROTEIN 1-3, **PIP1-3**	AT1G01620	3/3	Aquaporin, transmembrane water transporter at PM	TAIR (Boursiac et al., 2005)
VACUOLAR PROTON ATPase SUBUNIT A3, **VHA-A3**	AT4G39080	3/3	Vacuolar proton transport	TAIR (Dettmer et al., 2006)
VACUOLAR PROTON ATPase SUBUNIT D1, **VHA-D1**	AT3G28710	3/3	ATPase, V0/A0 complex, subunit C/D	TAIR
VACUOLAR PROTON ATPase SUBUNIT B2, **VHA-B2**	AT4G38510	3/1	Vacuolar proton transport	TAIR
AP-4 complex subunit mu, **AP4M**	AT4G24550	3/3	Clathrin adaptor complex, vacuolar sorting of storage proteins by interaction with VACUOLAR SORTING RECEPTOR1	TAIR (Fuji et al., 2016)
AP-2 complex subunit alpha-2, **ALPHAC-AD**	AT5G22780	3/1	Clathrin adaptor complex, endocytosis, vesicle transport	TAIR (Barth and Holstein, 2004)
Probable clathrin assembly protein	AT4G32285	2/3	Adaptor protein, membrane trafficking	TAIR
Coatomer subunit delta	AT5G05010	3/3	Protein transport, COPI vesicles	TAIR
SEC13A homolog, **SEC13A**	AT3G01340	3/2	COPII vesicle budding, protein transport	TAIR
Reticulon-like protein B5, **RTNLB5**	AT2G46170	3/2	ER-Golgi trafficking, vesicle formation and membrane morphogenesis	TAIR
FLOTTILIN-LIKE 1, **FLOT1**	AT5G25250	1/3	Hypoxia response, membrane invagination, clathrin-independent endocytosis	TAIR (Li et al., 2012)
Spatacsin carboxy terminus protein	AT4G39420	3/0	Associated with lysosomal function in mammals	(Khundadze et al., 2013)
ADP-RIBOSYLATION FACTOR A1E, **ARFA1E**	AT3G62290	3/2	ARF GTPase family, vesicle coating/ uncoating function	TAIR

*Protein names, AGI identifiers and number of replicates found under control or heat stress conditions are given. Short descriptions are based on annotations on TAIR, Uniprot or on literature. Proteins that were further characterized for their subcellular localization are highlighted in gray*.

a *In VPS2.2 Interactome*.

### Heat-Dependent Subcellular Localization of Interactome Candidates

In a next step, we selected proteins from the SKD1 interactome linked to membrane trafficking and RNA-associated processes and analyzed their subcellular localization before and after heat stress. The respective CDS were fused with YFP or mCH and expressed under the *35S CaMV* promoter. The selected membrane trafficking proteins included the 14-3-3-like protein GRF9 (Keicher et al., 2017), the WD-40 and putative vesicle coat protein SEC13A, the membrane microdomain protein FLOT1 (Li et al., 2012), and ESCRTIII associated proteins ISTL1 and CHMP1A. Additionally, we analyzed the ESCRTIII core and associated proteins SNF7.2, VPS24.1, and CHMP1B. The class of proteins linked to RNA-associated processes included the translation initiation factor eIF4B1 (Mayberry et al., 2009), the DEAD-box RNA helicases UAP56A (Kammel et al., 2013; Pfaff et al., 2018) and LOS4 (Gong et al., 2002, 2005), CML10, a family member protein of CML38 that regulates mRNP granule formation under hypoxia (Lokdarshi et al., 2016), the small nuclear mRNP SMF (Kanno et al., 2017), and the RNA Recognition Motif (RRM) -containing protein NTF2 (Parida et al., 2017).

**Figure 6:**
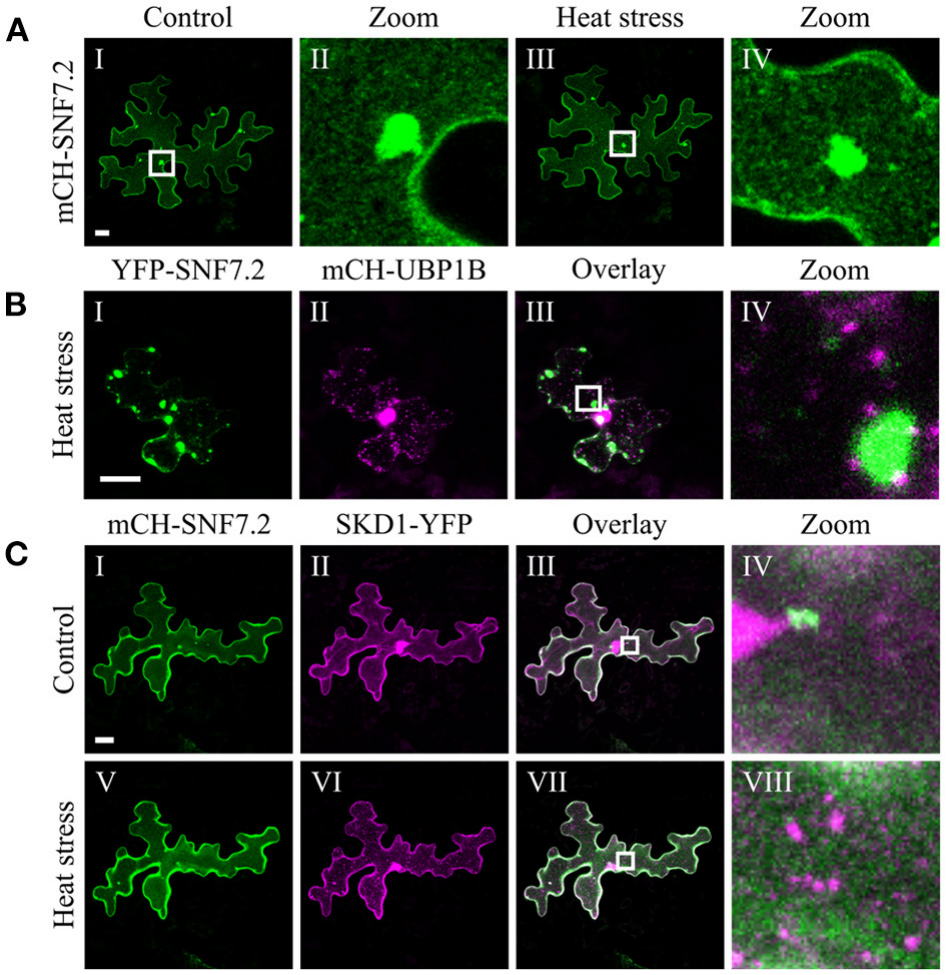
Subcellular localization of SNF7.2 before and after heat stress. Representative images of CLSM stacks (maximum projections) of leaf epidermal cells single or double transformed with constructs overexpressing (*35S* promoter) SNF7.2. The results are summarized in Table 3. The same cells were imaged before and after heat stress. For clarity, SNF7.2 is shown in green and UBP1b and SKD1 are depicted in magenta, independent of the fluorescent tag. Scale bar = 20 μm. **(A)** mCH-SNF7.2, **(B)** YFP-SNF7.2 with mCH-UBP1B, **(C)** mCH-SNF7.2 with SKD1-YFP.

**Figure 7:**
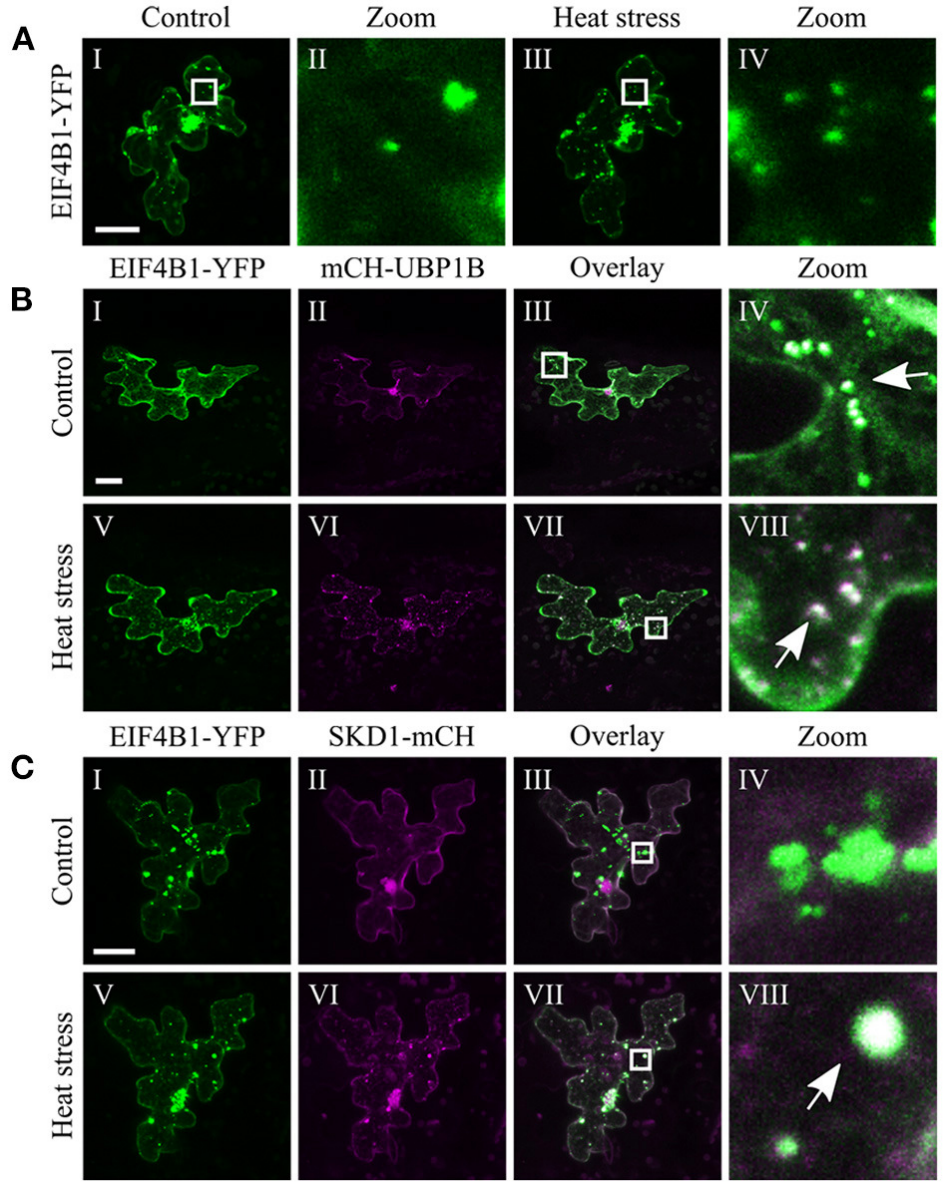
Subcellular localization of EIF4B1 before and after heat stress. Representative images of CLSM stacks (maximum projections) of leaf epidermal cells single or double transformed with constructs overexpressing (*35S* promoter) EIF4B1. The results are summarized in Table 3. The same cells were imaged before and after heat stress. Co-localizing structures are indicated by arrow heads. Scale bar = 20 μm. EIF4B1-YFP **(A)** alone, **(B)** with mCH-UBP1B, **(C)** with SKD1-mCH.

**Figure 8:**
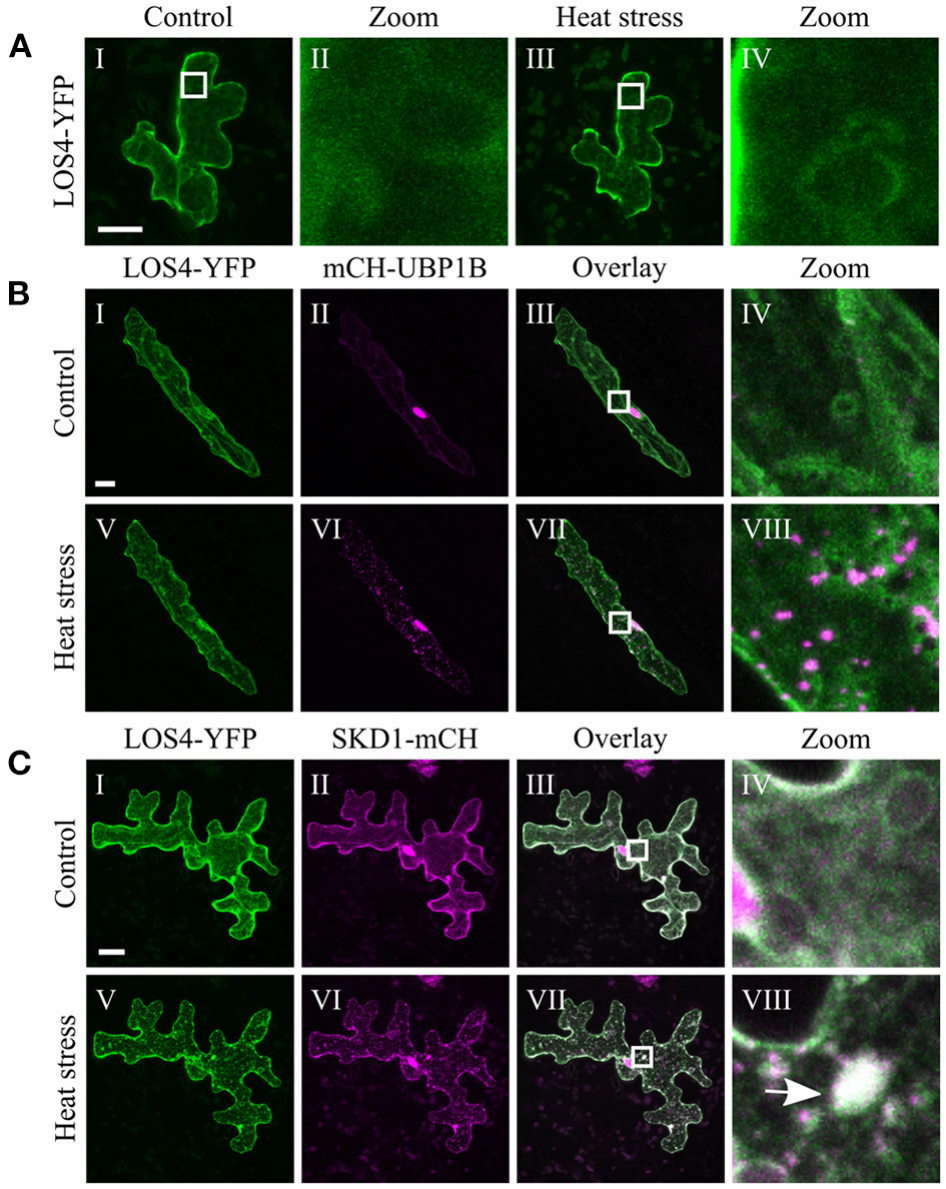
Subcellular localization of LOS4 before and after heat stress. Representative images of CLSM stacks (maximum projections) of leaf epidermal cells single or double transformed with constructs overexpressing (*35S* promoter) LOS4. The results are summarized in Table 3. The same cells were imaged before and after heat stress. Co-localizing structures are indicated by arrow heads. Scale bar = 20 μm. LOS4-YFP **(A)** alone, **(B)** with mCH-UBP1B, **(C)** with SKD1-mCH.

**Figure 9:**
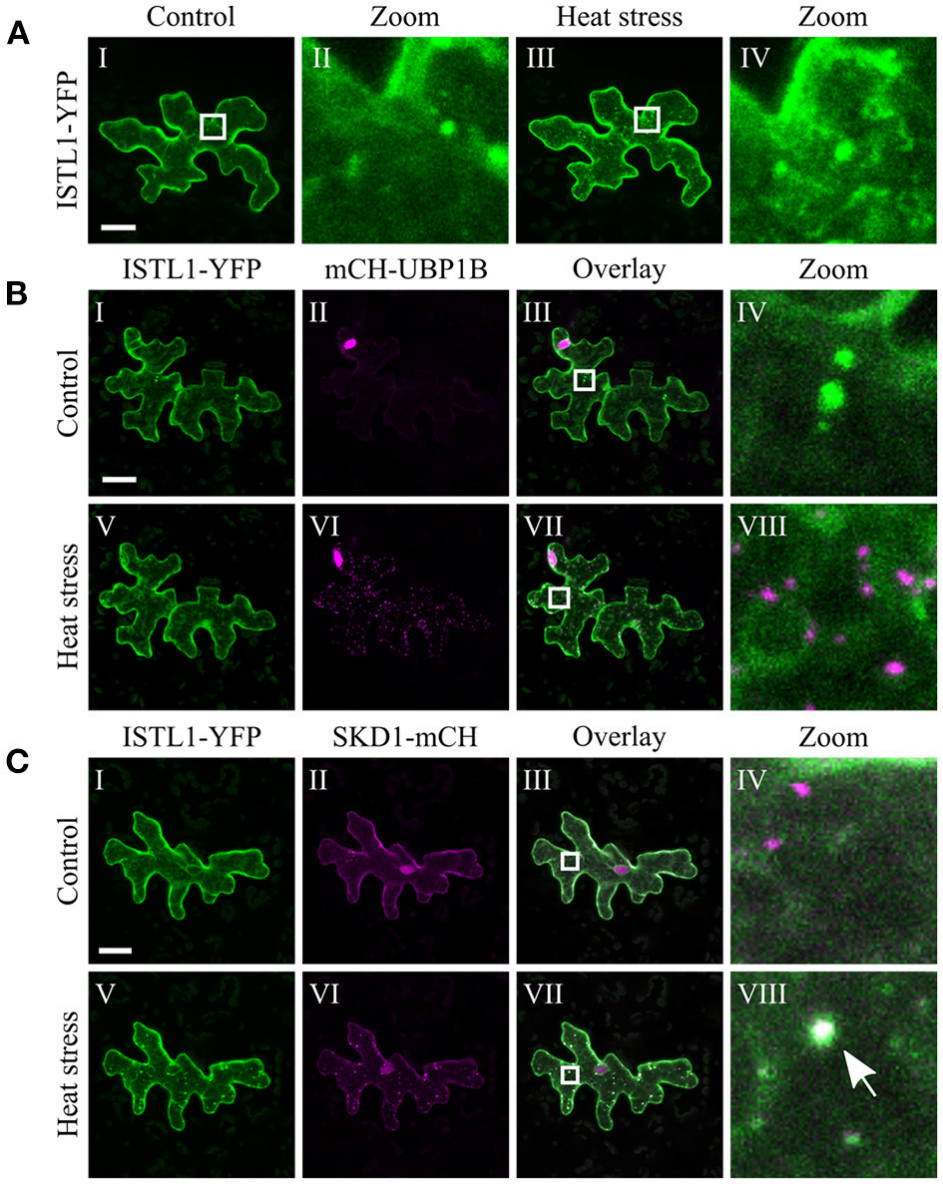
Subcellular localization of ISTL1 before and after heat stress. Representative images of CLSM stacks (maximum projections) of leaf epidermal cells single or double transformed with constructs overexpressing (*35S* promoter) ISTL1. The results are summarized in Table 3. The same cells were imaged before and after heat stress. Co-localizing structures are indicated by arrow heads. Scale bar = 20 μm. ISTL1-YFP **(A)** alone, **(B)** with mCH-UBP1B, **(C)** with SKD1-mCH.

The chosen proteins were analyzed for a granular or dot-like localization before and after heat treatment when transiently transformed alone, co-transformed with UBP1b or with SKD1 (representative pictures in Figures 6–9 and Supplementary Figures 6–15, summary in Table 3). From 14 analyzed proteins, only the ESCRTIII core protein SNF7.2 did not co-localize with SKD1 or UBP1B and was visible in the cytosol and dot-like structures independent of heat treatment (Figure 6). The other 13 proteins showed at least partial co-localization with SKD1 in granules after heat treatment. Among these, nine proteins were found in granules with UBP1B after heat stress, of which only EIF4B and NTF2 also co-localized with UBP1B in granules before heat stress (Figure 7 and Supplementary Figure 13). Two proteins which did not co-localize with UBP1B, LOS4, and UAP56A, were only in granules after heat when co-expressed with SKD1 (Figure 8 and Supplementary Figure 14). ESCRTIII proteins ISTL1 and VPS24.1 are also present in some dot-like or granular structures in the absence of heat stress (Figure 9 and Supplementary Figure 15).

**Table 3:** Summary of subcellular localization of ESCRTIII and SKD1 interactome proteins.

			**Membrane trafficking/ESCRTIII**								**RNA-associated processes**					
			**GRF9**	**SEC13A**	**FLOT1**	**ISTL1**	**SNF7.2**	**VPS24.1**	**CHMP1B**	**CHMP1A**	**EIF4B1**	**UAP56A**	**LOS4**	**CML10**	**SMF**	**NTF2**
Alone	Control	In dots?	–	–	–	+	+	+	–	–	+	–	–	+	–	+
	Heat stress	In dots?	+	+	+	+	+	+	+	+	+	–	–	+	+	+
With	Control	In dots?	–	–	–	+	n.d.	n.d.	n.d.	n.d.	+	–	–	+	–	+
UBP1B		Co-localization?	–	–	–	–	n.d.	n.d.	n.d.	n.d.	+	–	–	–	–	+
	Heat stress	In dots?	+	+	+	+	+	+	+	+	+	–	–	+	+	+
		Co-localization?	+	+	+	–	–	–	+	+	+	–	–	+	+	+
With	Control	In dots?	–	–	–	+	+	+	–	–	+	–	–	+	–	+
SKD1		Co-localization?	–	–	–	–	–	–	–	–	–	–	–	–	–	–
	Heat stress	In dots?	+	+	+	+	+	+	+	+	+	+	+	+	+	+
		Co-localization?	+	+	+	+	–	+	+	+	+	+	+	+	+	+

*Leaf epidermal cells were transiently transformed with constructs overexpressing (35S promoter) ESCRTIII or proteins identified in the SKD1 interactome in C- or N-terminal fusion with YFP or mCH. In co-localization studies, cells were additionally transformed with a construct overexpressing SKD1-YFP/mCH or YFP/mCH-UBP1B. The subcellular localization of proteins regarding the presence of dot-like or granular structures before and after heat stress was analyzed by CLSM. At least 3–7 cells were analyzed for each combination. Representative pictures are given in Supplementary Figures 6–15. n.d., not determined*.

Finally, localization before and after heat treatment was exemplarily analyzed for SEC13A and FLOT1 when transiently co-transformed with SKD1-AQ (Supplementary Figure 16). Both proteins co-localized with SKD1-AQ-marked dots and larger aggregates representing class E compartments.

## Discussion

### Heat Stress Dependent Localization of Membrane Transport Proteins to SGs Is Selective

The finding that SKD1 co-localizes with SG (UBP1b and PAB2) and P-body (DCP1 and DCP5) markers after heat stress raises the question, whether this is a general behavior of ESCRT proteins or other regulators of membrane transport processes. Our results show that several proteins, including GRF9, SEC13A, FLOT1, CHMP1B, and CHMP1A, show a similar localization behavior as SKD1. This co-localization does not appear to be mediated by the ATPase function of SKD1, as exemplarily shown for SEC13A and FLOT1. Also, our co-localization experiments with the membrane marker FM4-64 suggest that the co-localization of SKD1 with SGs is not due to an association with endosomal structures. Consistent with this, the ESCRTIII protein VPS24.1 and ISTL1, as well as the endosomal marker protein ARA7, labeled a population of dots distinct from the UBP1b-labeled dots after heat shock, indicating that not all ESCRT proteins are recruited to SGs. Among the tested SKD1 interactors involved in membrane trafficking, only SNF7.2 showed no co-localization with SKD1 or the SG marker UBP1B.

Taken together, our data show that SKD1 shares the localization behavior with a number of other proteins involved in membrane trafficking, but that heat stress dependent localization of membrane transport proteins to SGs is also selective.

### SKD1 Interactions With Membrane- and RNA-Associated Proteins Are Not Stress-Induced

Our comparison of the interactome revealed that only about 10% of the SKD1 interactors were heat stress specific. However, when interpreting interactome data of proteins located to endosomal structures and mRNA granules, it has to be considered that certain classes of proteins may escape the detection. Proteins that are tightly linked to membranes or that interact only weakly with the bait, as discussed for RNA granules (Jain et al., 2016; Protter and Parker, 2016), may not be found in the interactome. Considering this transient nature of protein-protein interactions, fixation prior to cell lysis might be necessary to highlight treatment-specific differences in interactor abundances in future studies.

The heat stress specific interactors found in this study included proteins involved in protein folding. Comparing the interactome of SKD1 with those from the two SG proteins RBP45B and CML38 (Lokdarshi et al., 2016; Muthuramalingam et al., 2017), we noticed that the three data sets share this group of proteins, most of them being heat shock proteins (HSPs) and TCP-1 chaperones. TCP-1 proteins, also known as CCT complex proteins, form cytosolic ring complexes and assist in the protein folding of various cytosolic proteins (Leitner et al., 2012). They have been identified in purified yeast SGs and were shown to negatively regulate SG formation in mutant studies (Jain et al., 2016). The presence of several TCP-1 proteins in the SKD1, RBP45B, and CML38 interactome might hint toward a similar role of this class of chaperones in plant mRNP granules.

SKD1 interactors found under both, normal conditions and heat stress, included GO terms related to the known function of SKD1, such as intracellular protein transport, vesicle-mediated transport, and protein targeting. Notably, the interactors of the membrane transport class and the RNA-associated proteins were found under normal and stress conditions suggesting that the associations with SG/P-bodies are not stress-induced.

### Possible Functional Relevance of SKD1 in Stress-Dependent Recruitment to mRNPs

The stability of SGs is ATP dependent, and specific ATP-dependent remodeling complexes such as the chaperonin-containing T (CCT) complex or the RNA helicase complex RuvB-like (Rvb) actively modulate the assembly and disassembly of SGs. For the AAA-ATPase Cell Division Cycle 48 (Cdc48), it was shown that it removes ubiquitinated proteins from SGs, thereby contributing to their disassembly (Buchan et al., 2013). Given that SKD1 is an ATPase, it would be possible that SKD1 is involved in SG assembly and disassembly. This, however, is not supported by our results, as the ATPase-defective SKD1 version SKD1-AQ has no inhibitory effect on mRNP granule formation.

Another possible function of SKD1 could be the recruitment of other proteins to mRNPs. In support of this idea, SKD1 promotes the localization of UAP56A and LOS4 to cytoplasmic foci in response to heat stress.

An explanation for the recruitment of SKD1 and other membrane trafficking proteins into mRNPs might be that this re-localization functions as a quick and reversible way to inhibit trafficking processes during heat stress. The concept that mRNP granules regulate cellular processes by recruiting key signaling factors into granules during cellular stress has been put forward before (Protter and Parker, 2016). A well-described example is the recruitment of TARGET OF RAPAMYCIN COMPLEX 1 (TORC1), a regulator of the metabolic state of eukaryotic cells (Kedersha et al., 2013). Usually, TORC1 is active in lysosomal/vacuolar membranes. Stress treatment leads to an inhibition of TORC1 signaling, correlated with a localization to SGs (Takahara and Maeda, 2012; Wippich et al., 2013). Another recent example is the sequestration of the senescence-inducing factor PLASMINOGEN ACTIVATOR INHIBITOR 1 (PAI-1) into SGs during prolonged cellular stress to prevent the onset of senescence in proliferating human cells (Omer et al., 2018). If this principle was the case for SKD1, one would expect that the transport of membrane proteins targeted for degradation in the vacuole would be stopped by heat shock. In support of this, previous studies in yeast have shown that heat treatment delays FM4-64 staining of late endosomal structures and the vacuolar membrane, suggesting a specific inhibition of late trafficking events (Meaden et al., 1999). A similar observation was made in a study investigating the function of the Huntington's disease protein HUNTINGTIN during heat stress (Nath et al., 2015). The authors showed that heat stress induces the rapid association of HUNTINGTIN with early endosomes, which causes the arrest of endosome maturation.

Taken together, former studies and our data presented here suggest a link of SKD1 and mRNPs. Additional studies are needed to unravel possible relevance of the stress-dependent, selective association with other proteins.

## Data Availability Statement

The data presented in the study are deposited in the ProteomeXchange Consortium via the PRIDE (Perez-Riverol et al., 2019) partner repository with the dataset identifier PXD025028.

## Author Contributions

HW, MJ, LS, and MH designed this study. HW, LS, and MH wrote the manuscript. HW and EK performed the research. HW analyzed the data. MH directed the project. All authors contributed to the article and approved the submitted version.

## Conflict of Interest

The authors declare that the research was conducted in the absence of any commercial or financial relationships that could be construed as a potential conflict of interest.

## Supplementary Material

Supplementary Figure 1 FM4-64 staining of *35S::GFP-SKD1* roots. *35S::GFP-SKD1* roots of 5 or 7d old seedlings were stained with 50 μM FM4-64 in $\frac{1}{2}$ MS liquid medium for 5 min. Cells were incubated at room temperature for 2 h and then subjected to heat treatment. Roots were analyzed by CLSM after heat treatment and single planes of root epidermal cells of the proximal transformation zone/early elongation zone are depicted. Scale bar = 20 μm.

Supplementary Figure 2 Co-expression of mCH-UBP1B with free YFP or GFP-SKD1. Representative images of epidermal cells of Col-0 **(A)** or *35S::GFP-SKD1* **(B)** rosette leaves that were transiently transformed by particle bombardment with a construct overexpressing mCHERRY-UBP1B. In **(A)**, cells were additionally co-transformed with a construct overexpressing free YFP. Cells were imaged with the same laser intensities before and after heat treatment by CLSM. Maximum projections of CLSM stacks are depicted. Scale bar = 20 μm.

Supplementary Figure 3 Expression of SKD1 in the autophagy-deficient mutant *atg5-1* and in monodansylcadaverine-stained leaves. Representative images of CLSM stacks (maximum projections) of leaf epidermal cells. Scale bar = 20 μm. **(A)** *atg5-1* mutant double transformed with constructs overexpressing (*35S* promoter) SKD1-YFP and CFP-UBP1B. The same cells were imaged before and after heat stress. Co-localizing structures are indicated by arrows. **(B)** Monodansylcadaverine (MDC)-stained Col-0 leaves transformed with a construct overexpressing (*35S* promoter) SKD1-mCH. Arrows indicate MDC-stained vesicles.

Supplementary Figure 4 Co-expression of SKD1-AQ and UBP1B and pictures of the positive controls of the co-localization quantification. Representative images of transiently transformed epidermal cells co-expressing (*35S* promoter) YFP-UBP1B and mCH-UBP1B **(A)**, YFP-UBP1B and mCH-PAB2 **(B)**, or YFP-SKD1-AQ and mCH-UBP1B **(C)**. The cells were imaged after heat stress by CLSM microscopy and maximum projections are depicted. Arrows indicate co-localizing structures in zoom of overlays (IV). Scale bar = 20 μm.

Supplementary Figure 5 PANTHER GO enrichment analysis of SKD1 shared interactors. The PANTHER classification System was used to evaluate the interactome candidates of the class “SKD1 shared interactors.” All categories of the PANTHER GO-Slim Biological Process **(A)** and Cellular Component **(B)**, which were significantly enriched in the SKD1 shared interactor list (*q* ≤ 0.05), are indicated in the histogram. The fold enrichment of a category compared to the whole *A. thaliana* genome and the number of proteins in each category are depicted.

Supplementary Figure 6 Subcellular localization of GRF9 before and after heat stress. Representative images of CLSM stacks (maximum projections) of leaf epidermal cells single or double transformed with constructs overexpressing (*35S* promoter) ESCRTIII or proteins identified in the SKD1 interactome. For each combination, at least 3–7 cells were analyzed and the results are summarized in Table 3. The same cells were imaged before and after heat stress. For clarity, the ESCRTIII and interactome proteins are depicted in green and UBP1b and SKD1 are depicted in magenta, independent of the fluorescent tag. Co-localizing structures are indicated by arrow heads. Scale bar = 20 μm. YFP-GRF9 **(A)** alone, **(B)** with mCH-UBP1B, **(C)** with SKD1-mCH.

Supplementary Figure 7 Subcellular localization of SEC13A before and after heat stress. Representative images of CLSM stacks (maximum projections) of leaf epidermal cells single or double transformed with constructs overexpressing (*35S* promoter) ESCRTIII or proteins identified in the SKD1 interactome. For each combination, at least 3–7 cells were analyzed and the results are summarized in Table 3. The same cells were imaged before and after heat stress. For clarity, the ESCRTIII and interactome proteins are depicted in green and UBP1b and SKD1 are depicted in magenta, independent of the fluorescent tag. Co-localizing structures are indicated by arrow heads. Scale bar = 20 μm. SEC13A-YFP **(A)** alone, **(B)** with mCH-UBP1B, **(C)** with SKD1-mCH.

Supplementary Figure 8 Subcellular localization of FLOT1 before and after heat stress. Representative images of CLSM stacks (maximum projections) of leaf epidermal cells single or double transformed with constructs overexpressing (*35S* promoter) ESCRTIII or proteins identified in the SKD1 interactome. For each combination, at least 3–7 cells were analyzed and the results are summarized in Table 3. The same cells were imaged before and after heat stress. For clarity, the ESCRTIII and interactome proteins are depicted in green and UBP1b and SKD1 are depicted in magenta, independent of the fluorescent tag. Co-localizing structures are indicated by arrow heads. Scale bar = 20 μm. YFP-FLOT1 **(A)** alone. **(B)** with mCH-UBP1B, **(C)** with SKD1-mCH.

Supplementary Figure 9 Subcellular localization of CHMP1B before and after heat stress. Representative images of CLSM stacks (maximum projections) of leaf epidermal cells single or double transformed with constructs overexpressing (*35S* promoter) ESCRTIII or proteins identified in the SKD1 interactome. For each combination, at least 3–7 cells were analyzed and the results are summarized in Table 3. The same cells were imaged before and after heat stress. For clarity, the ESCRTIII and interactome proteins are depicted in green and UBP1b and SKD1 are depicted in magenta, independent of the fluorescent tag. Co-localizing structures are indicated by arrow heads. Scale bar = 20 μm. mCH-CHMP1B **(A)** alone, **(B)** with YFP-UBP1B, **(C)** with SKD1-YFP.

Supplementary Figure 10 Subcellular localization of CHMP1A before and after heat stress. Representative images of CLSM stacks (maximum projections) of leaf epidermal cells single or double transformed with constructs overexpressing (*35S* promoter) ESCRTIII or proteins identified in the SKD1 interactome. For each combination, at least 3–7 cells were analyzed and the results are summarized in Table 3. The same cells were imaged before and after heat stress. For clarity, the ESCRTIII and interactome proteins are depicted in green and UBP1b and SKD1 are depicted in magenta, independent of the fluorescent tag. Co-localizing structures are indicated by arrow heads. Scale bar = 20 μm. YFP-CHMP1A **(A)** alone, **(B)** with mCH-UBP1B, **(C)** mCH-CHMP1A with SKD1-YFP.

Supplementary Figure 11 Subcellular localization of CML10 before and after heat stress. Representative images of CLSM stacks (maximum projections) of leaf epidermal cells single or double transformed with constructs overexpressing (*35S* promoter) ESCRTIII or proteins identified in the SKD1 interactome. For each combination, at least 3–7 cells were analyzed and the results are summarized in Table 3. The same cells were imaged before and after heat stress. For clarity, the ESCRTIII and interactome proteins are depicted in green and UBP1b and SKD1 are depicted in magenta, independent of the fluorescent tag. Co-localizing structures are indicated by arrow heads. Scale bar = 20 μm. CML10-YFP **(A)** alone, **(B)** with mCH-UBP1B, **(C)** with SKD1-mCH.

Supplementary Figure 12 Subcellular localization of SMF before and after heat stress. Representative images of CLSM stacks (maximum projections) of leaf epidermal cells single or double transformed with constructs overexpressing (*35S* promoter) ESCRTIII or proteins identified in the SKD1 interactome. For each combination, at least 3–7 cells were analyzed and the results are summarized in Table 3. The same cells were imaged before and after heat stress. For clarity, the ESCRTIII and interactome proteins are depicted in green and UBP1b and SKD1 are depicted in magenta, independent of the fluorescent tag. Co-localizing structures are indicated by arrow heads. Scale bar = 20 μm. SMF-YFP **(A)** alone, **(B)** with mCH-UBP1B, **(C)** with SKD1-mCH. SMF-YFP was usually cytosolic under control conditions (**A**I,**C**I) and in granules after heat stress (**A**III,**B**IX,**C**V). Occasionally, SMF-YFP was visible in larger aggregates in the cell (**B**I), which inhibited granule formation (**B**V,VI), but still allowed co-localization with mCH-UBP1B (**B**VIII). When SMF-YFP was not in aggregates before heat stress, normal appearing granules were formed after heat stress (**B**IX–XII).

Supplementary Figure 13 Subcellular localization of NTF2 before and after heat stress. Representative images of CLSM stacks (maximum projections) of leaf epidermal cells single or double transformed with constructs overexpressing (*35S* promoter) ESCRTIII or proteins identified in the SKD1 interactome. For each combination, at least 3–7 cells were analyzed and the results are summarized in Table 3. The same cells were imaged before and after heat stress. For clarity, the ESCRTIII and interactome proteins are depicted in green and UBP1b and SKD1 are depicted in magenta, independent of the fluorescent tag. Co-localizing structures are indicated by arrow heads. Scale bar = 20 μm. **(A)** YFP-NTF2, **(B)** with mCH-UBP1B, **(C)** with SKD1-mCH.

Supplementary Figure 14 Subcellular localization of UAP56A before and after heat stress. Representative images of CLSM stacks (maximum projections) of leaf epidermal cells single or double transformed with constructs overexpressing (*35S* promoter) ESCRTIII or proteins identified in the SKD1 interactome. For each combination, at least 3–7 cells were analyzed and the results are summarized in Table 3. The same cells were imaged before and after heat stress. For clarity, the ESCRTIII and interactome proteins are depicted in green and UBP1b and SKD1 are depicted in magenta, independent of the fluorescent tag. Co-localizing structures are indicated by arrow heads. Scale bar = 20 μm. YFP-UAP56A **(A)** alone, **(B)** with mCH-UBP1B, **(C)** with SKD1-mCH.

Supplementary Figure 15 Subcellular localization of VPS24.1 before and after heat stress. Representative images of CLSM stacks (maximum projections) of leaf epidermal cells single or double transformed with constructs overexpressing (*35S* promoter) ESCRTIII or proteins identified in the SKD1 interactome. For each combination, at least 3–7 cells were analyzed and the results are summarized in Table 3. The same cells were imaged before and after heat stress. For clarity, the ESCRTIII and interactome proteins are depicted in green and UBP1b and SKD1 are depicted in magenta, independent of the fluorescent tag. Co-localizing structures are indicated by arrow heads. Scale bar = 20 μm. **(A)** VPS24.1-YFP, **(B)** VPS24.1-mCH with YFP-UBP1B, **(C)** with SKD1-YFP.

Supplementary Figure 16 Subcellular localization of exemplary interactome candidates and SKD1-AQ before and after heat stress. Representative images of CLSM stacks (maximum projections) of leaf epidermal cells triple transformed with constructs overexpressing (*35S* promoter) proteins identified in the SKD1 interactome (*UBQ10* promoter) SKD1-AQ and (*35S* promoter) UBP1B. Distinct, dot-like co-localizing structures are indicated by arrow heads. Scale bar = 20 μm. **(A)** SEC13A-YFP with mCH-SKD1-AQ and CFP-UBP1B, **(B)** YFP-FLOT1 with mCH-SKD1-AQ and CFP-UBP1B.

Supplementary Table 1 Raw table of the proteins identified by LC-MS/MS analysis. All proteins identified in the interactome studies are listed. Protein IDs (ARATH), protein names and/or gene names are indicated. The columns “#values” indicate the number of replicates, in which a protein was identified (at least 2 peptides). The samples of the *35S::YFP* control line are indicated as “cntrl,” the samples of the *35S::GFP-SKD1* line are indicated as “SKD1.” Label-free quantifications (LFQs), the number of identified peptides and sequence coverage are indicated for each replicate. The iBAQ values for each protein were calculated across all samples. The sample numbers 1–3 refer to the untreated “cntrl,” 4–6 to “cntrl heat,” 7–9 to “SKD1,” and 10–12 to “SKD1 heat.” Significant differences in protein abundances (untreated vs. heat) were tested when a protein was identified in at least two replicates of each condition and -log *p*-values and adjusted q-values are given.

Supplementary Table 2 List of filtered interactome candidates. Proteins that were identified in at least three replicates of one condition of the *35S::GFP-SKD1* samples and were not present in any of the *35S::YFP* samples. Names, Protein IDs, and ATG gene identifier are given. Proteins of the “SKD1 control” class were present in three replicates of the untreated samples and in none or one of the heat-treated samples. Proteins in the “SKD1 Heat stress” class were present in none or one replicate of the untreated samples and in all three replicates of the heat-treated samples. Annotations and short descriptions are based on TAIR annotations and available literature. Proteins that are involved in membrane trafficking processes are labeled in orange, proteins that are involved in RNA-associated processes are labeled in lilac. The column “in other interactome” indicates, if an identified SKD1 interactor was found in an *A. thaliana* interactome of the SG proteins CML38 (Lokdarshi et al., 2016), RBP45B (Muthuramalingam et al., 2017), or the ESCRTIII protein VPS2.2 (Ibl et al., 2012).

## References

[B1] Aizer A. Kalo A. Kafri P. Shraga A. Ben-Yishay R. Jacob A.. (2014). Quantifying mRNA targeting to P-bodies in living human cells reveals their dual role in mRNA decay and storage. J. Cell Sci. 127, 4443–4456. 10.1242/jcs.152975 25128566

[B2] Anderson P. Kedersha N. (2008). Stress granules: the Tao of RNA triage. Trends Biochem. Sci. 33, 141–150. 10.1016/j.tibs.2007.12.003 18291657

[B3] Azmi I. Davies B. Dimaano C. Payne J. Eckert D. Babst M.. (2006). Recycling of ESCRTs by the AAA-ATPase Vps4 is regulated by a conserved VSL region in Vta 1. J. Cell Biol. 172, 705–717. 10.1083/jcb.200508166 16505166 PMC2063703

[B4] Babst M. Wendland B. Estepa E. J. Emr S. D. (1998). The Vps4p AAA ATPase regulates membrane association of a Vps protein complex required for normal endosome function. EMBO J. 17, 2982–2993. 10.1093/emboj/17.11.2982 9606181 PMC1170638

[B5] Barth M. Holstein S. E. H. (2004). Identification and functional characterization of Arabidopsis AP180, a binding partner of plant αC-adaptin. J. Cell Sci. 117, 2051–2062. 10.1242/jcs.01062 15054111

[B6] Bhasin H. Hülskamp M. (2017). ANGUSTIFOLIA, a plant homolog of CtBP/BARS localizes to stress granules and regulates their formation. Front. Plant Sci. 8:1004. 10.3389/fpls.2017.01004 28659951 PMC5469197

[B7] Bhattacharyya S. N. Habermacher R. Martine U. Closs E. I. Filipowicz W. (2006). Relief of microRNA-mediated translational repression in human cells subjected to stress. Cell 125, 1111–1124. 10.1016/j.cell.2006.04.031 16777601

[B8] Bolte S. Cordelières F. P. (2006). A guided tour into subcellular colocalization analysis in light microscopy. J. Microsc. 224, 213–232. 10.1111/j.1365-2818.2006.01706.x 17210054

[B9] Bouché N. Scharlat A. Snedden W. Bouchez D. Fromm H. (2002). A novel family of calmodulin-binding transcription activators in multicellular organisms. J. Biol. Chem. 277, 21851–21861. 10.1074/jbc.M200268200 11925432

[B10] Boursiac Y. Chen S. Luu D. T. Sorieul M. Van Den Dries N. Maurel C. (2005). Early effects of salinity on water transport in Arabidopsis roots. Molecular and cellular features of aquaporin expression. Plant Physiol. 139, 790–805. 10.1104/pp.105.065029 16183846 PMC1255996

[B11] Brengues M. Teixeira D. Parker R. (2005). Cell biology: movement of eukaryotic mRNAs between polysomes and cytoplasmic processing bodies. Science 310, 486–489. 10.1126/science.1115791 16141371 PMC1863069

[B12] Buchan J. R. Kolaitis R. M. Taylor J. P. Parker R. (2013). Eukaryotic stress granules are cleared by autophagy and Cdc48/VCP function. Cell 153:1461. 10.1016/j.cell.2013.05.037 23791177 PMC3760148

[B13] Buchan J. R. Parker R. (2009). Eukaryotic stress granules: the Ins and outs of translation. Mol. Cell 36, 932–941. 10.1016/j.molcel.2009.11.020 20064460 PMC2813218

[B14] Buono R. A. Paez-Valencia J. Miller N. D. Goodman K. Spitzer C. Spalding E. P.. (2016). Role of SKD1 regulators LIP5 and IST1-LIKE1 in endosomal sorting and plant development. Plant Physiol. 171, 251–264. 10.1104/pp.16.00240 26983994 PMC4854716

[B15] Cho K. M. Nguyen H. T. K. Kim S. Y. Shin J. S. Cho D. H. Hong S. B.. (2016). CML10, a variant of calmodulin, modulates ascorbic acid synthesis. New Phytol. 209, 664–678. 10.1111/nph.13612 26315131

[B16] Cui P. Chen T. Qin T. Ding F. Wang Z. Chen H.. (2016). The RNA polymerase II C-terminal domain phosphatase-like protein FIERY2/CPL1 interacts with eIF4AIII and is essential for nonsense-mediated mRNA decay in Arabidopsis. Plant Cell 28, 770–785. 10.1105/tpc.15.00771 26887918 PMC4826008

[B17] Cui Y. He Y. Cao W. Gao J. Jiang L. (2018). The multivesicular body and autophagosome pathways in plants. Front. Plant Sci. 9:1837. 10.3389/fpls.2018.01837 30619408 PMC6299029

[B18] Dettmer J. Hong-Hermesdorf A. Stierhof Y. D. Schumacher K. (2006). Vacuolar H+-ATPase activity is required for endocytic and secretory trafficking in Arabidopsis. Plant Cell 18, 715–730. 10.1105/tpc.105.037978 16461582 PMC1383645

[B19] Dong A. Liu Z. Zhu Y. Yu F. Li Z. Cao K.. (2005). Interacting proteins and differences in nuclear transport reveal specific functions for the NAP1 family proteins in plants. Plant Physiol. 138, 1446–1456. 10.1104/pp.105.060509 15980199 PMC1176416

[B20] Dunn K. W. Kamocka M. M. McDonald J. H. (2011). A practical guide to evaluating colocalization in biological microscopy. Am. J. Physiol. Cell Physiol. 300, C723–C742. 10.1152/ajpcell.00462.2010 21209361 PMC3074624

[B21] Ewan R. Pangestuti R. Thornber S. Craig A. Carr C. O'Donnell L.. (2011). Deubiquitinating enzymes AtUBP12 and AtUBP13 and their tobacco homologue NtUBP12 are negative regulators of plant immunity. New Phytol. 191, 92–106. 10.1111/j.1469-8137.2011.03672.x 21388379

[B22] Feys B. J. Wiermer M. Bhat R. A. Moisan L. J. Medina-Escobar N. Neu C.. (2005). Arabidopsis senescence-associated gene101 stabilizes and signals within an enhanced disease susceptibility1 complex in plant innate immunity. Plant Cell 17, 2601–2613. 10.1105/tpc.105.033910 16040633 PMC1197438

[B23] Fuji K. Shirakawa M. Shimono Y. Kunieda T. Fukao Y. Koumoto Y.. (2016). The adaptor complex AP-4 regulates vacuolar protein sorting at the trans-golgi network by interacting with vacuolar sorting receptor11. Plant Physiol. 170, 211–219. 10.1104/pp.15.00869 26546666 PMC4704568

[B24] Gao C. Zhuang X. Shen J. Jiang L. (2017). Plant ESCRT complexes: moving beyond endosomal sorting. Trends Plant Sci. 22, 986–998. 10.1016/j.tplants.2017.08.003 28867368

[B25] Geldner N. Dénervaud-Tendon V. Hyman D. L. Mayer U. Stierhof Y. D. Chory J. (2009). Rapid, combinatorial analysis of membrane compartments in intact plants with a multicolor marker set. Plant J. 59, 169–178. 10.1111/j.1365-313X.2009.03851.x 19309456 PMC4854200

[B26] Gong Z. Dong C. H. Lee H. Zhu J. Xiong L. Gong D.. (2005). A DEAD box RNA helicase is essential for mRNA export and important for development and stress responses in Arabidopsis. Plant Cell 17, 256–267. 10.1105/tpc.104.027557 15598798 PMC544503

[B27] Gong Z. Lee H. Xiong L. Jagendorf A. Stevenson B. Zhu J. K. (2002). RNA helicase-like protein as an early regulator of transcription factors for plant chilling and freezing tolerance. Proc. Natl. Acad. Sci. U.S.A. 99, 11507–11512. 10.1073/pnas.172399299 12165572 PMC123286

[B28] Haas T. J. Sliwinski M. K. Martínez D. E. Preuss M. Ebine K. Ueda T.. (2007). The Arabidopsis AAA ATPase SKD1 is involved in multivesicular endosome function and interacts with its positive regulator LYST-INTERACTING PROTEIN5. Plant Cell 19, 1295–1312. 10.1105/tpc.106.049346 17468262 PMC1913750

[B29] Hao L. Liu J. Zhong S. Gu H. Qu L. J. (2016). AtVPS41-mediated endocytic pathway is essential for pollen tube-stigma interaction in Arabidopsis. Proc. Natl. Acad. Sci. U.S.A. 113, 6307–6312. 10.1073/pnas.1602757113 27185920 PMC4896696

[B30] Hartmann C. Chami M. Zachariae U. de Groot B. L. Engel A. Grütter M. G. (2008). Vacuolar protein sorting: two different functional states of the AAA-ATPase Vps4p. J. Mol. Biol. 377, 352–363. 10.1016/j.jmb.2008.01.010 18272179

[B31] Havé M. Balliau T. Cottyn-Boitte B. Dérond E. Cueff G. Soulay F.. (2018). Increases in activity of proteasome and papain-like cysteine protease in Arabidopsis autophagy mutants: back-up compensatory effect or cell-death promoting effect? J. Exp. Bot. 69, 1369–1385. 10.1093/jxb/erx482 29281085 PMC6037082

[B32] Huotari J. Helenius A. (2011). Endosome maturation. EMBO J. 30, 3481–3500. 10.1038/emboj.2011.286 21878991 PMC3181477

[B33] Hurley J. H. Hanson P. I. (2010). Membrane budding and scission by the ESCRT machinery: it's all in the neck. Nat. Rev. Mol. Cell Biol. 11, 556–566. 10.1038/nrm2937 20588296 PMC2922035

[B34] Ibl V. Csaszar E. Schlager N. Neubert S. Spitzer C. Hauser M. T. (2012). Interactome of the plant-specific ESCRT-III component AtVPS2.2 in *Arabidopsis thaliana*. J. Proteome Res. 11, 397–411. 10.1021/pr200845n 22010978 PMC3252797

[B35] Jain S. Wheeler J. R. Walters R. W. Agrawal A. Barsic A. Parker R. (2016). ATPase-modulated stress granules contain a diverse proteome and substructure. Cell 164, 487–498. 10.1016/j.cell.2015.12.038 26777405 PMC4733397

[B36] Jeong S. Y. Rose A. Joseph J. Dasso M. Meier I. (2005). Plant-specific mitotic targeting of RanGAP requires a functional WPP domain. Plant J. 42, 270–282. 10.1111/j.1365-313X.2005.02368.x 15807788

[B37] Kammel C. Thomaier M. Sørensen B. B. Schubert T. Längst G. Grasser M.. (2013). Arabidopsis DEAD-Box RNA helicase UAP56 interacts with Both RNA and DNA as well as with mRNA export factors. PLoS ONE 8:e60644. 10.1371/journal.pone.0060644 23555998 PMC3608606

[B38] Kanno T. Lin W. D. Fu J. L. Matzke A. J. M. Matzke M. (2017). A genetic screen implicates a CWC16/Yju2/CCDC130 protein and SMU1 in alternative splicing in *Arabidopsis thaliana*. RNA 23, 1068–1079. 10.1261/rna.060517.116 28373290 PMC5473141

[B39] Karampelias M. Neyt P. De Groeve S. Aesaert S. Coussens G. Rolcík J.. (2016). ROTUNDA3 function in plant development by phosphatase 2A-mediated regulation of auxin transporter recycling. Proc. Natl. Acad. Sci. U.S.A. 113, 2768–2773. 10.1073/pnas.1501343112 26888284 PMC4791031

[B40] Kedersha N. Ivanov P. Anderson P. (2013). Stress granules and cell signaling: more than just a passing phase? Trends Biochem. Sci. 38, 494–506. 10.1016/j.tibs.2013.07.004 24029419 PMC3832949

[B41] Kedersha N. Stoecklin G. Ayodele M. Yacono P. Lykke-Andersen J. Fitzler M. J.. (2005). Stress granules and processing bodies are dynamically linked sites of mRNP remodeling. J. Cell Biol. 169, 871–884. 10.1083/jcb.200502088 15967811 PMC2171635

[B42] Kedersha N. L. Gupta M. Li W. Miller I. Anderson P. (1999). RNA-binding Proteins TIA-1 and TIAR Link the Phosphorylation of eIF-2 to the Assembly of Mammalian Stress Granules. Available online at: http://www.jcb.org (accessed March 18, 2021). 10.1083/jcb.147.7.1431 PMC2174242 10613902

[B43] Keicher J. Jaspert N. Weckermann K. Möller C. Throm C. Kintzi A.. (2017). Arabidopsis 14-3-3 epsilon members contribute to polarity of PIN auxin carrier and auxin transport-related development. Elife 6:e24336. 10.7554/eLife.24336.021 28422008 PMC5397284

[B44] Khundadze M. Kollmann K. Koch N. Biskup C. Nietzsche S. Zimmer G.. (2013). A hereditary spastic paraplegia mouse model supports a role of ZFYVE26/SPASTIZIN for the endolysosomal system. PLoS Genet. 9:e1003988. 10.1371/journal.pgen.1003988 24367272 PMC3868532

[B45] Kidokoro S. Yoneda K. Takasaki H. Takahashi F. Shinozaki K. Yamaguchi-Shinozaki K. (2017). Different cold-signaling pathways function in the responses to rapid and gradual decreases in temperature. Plant Cell 29, 760–774. 10.1105/tpc.16.00669 28351986 PMC5435423

[B46] Kim S. J. Bassham D. C. (2011). Tno1 is involved in salt tolerance and vacuolar trafficking in arabidopsis. Plant Physiol. 156, 514–526. 10.1104/pp.110.168963 21521696 PMC3177255

[B47] Lambermon M. H. L. Simpson G. G. Wieczorek Kirk D. A. Hemmings-Mieszczak M. Klahre U. Filipowicz W. (2000). UBP1, a novel hnRNP-like protein that functions at multiple steps of higher plant nuclear pre-mRNA maturation. EMBO J. 19, 1638–1649. 10.1093/emboj/19.7.1638 10747031 PMC310232

[B48] Landsberg M. J. Vajjhala P. R. Rothnagel R. Munn A. L. Hankamer B. (2009). Three-dimensional structure of AAA ATPase Vps4: advancing structural insights into the mechanisms of endosomal sorting and enveloped virus budding. Structure 17, 427–437. 10.1016/j.str.2008.12.020 19278657

[B49] Lee G. J. Eun J. S. Myong H. L. Hwang I. (2004). The Arabidopsis Rab5 homologs Rha1 and Ara7 localize to the prevacuolar compartment. Plant Cell Physiol. 45, 1211–1220. 10.1093/pcp/pch142 15509844

[B50] Leitner A. Joachimiak L. A. Bracher A. Mönkemeyer L. Walzthoeni T. Chen B.. (2012). The molecular architecture of the eukaryotic chaperonin TRiC/CCT. Structure 20, 814–825. 10.1016/j.str.2012.03.007 22503819 PMC3350567

[B51] Li R. Liu P. Wan Y. Chen T. Wang Q. Mettbach U.. (2012). A membrane microdomain-associated protein, Arabidopsis Flot1, is involved in a clathrin-independent endocytic pathway and is required for seedling development. Plant Cell 24, 2105–2122. 10.1105/tpc.112.095695 22589463 PMC3442590

[B52] Lokdarshi A. Craig Conner W. McClintock C. Li T. Roberts D. M. (2016). Arabidopsis CML38, a calcium sensor that localizes to ribonucleoprotein complexes under hypoxia stress. Plant Physiol. 170, 1046–1059. 10.1104/pp.15.01407 26634999 PMC4734562

[B53] Lottridge J. M. Flannery A. R. Vincelli J. L. Stevens T. H. (2006). Vta1p and Vps46p regulate the membrane association and ATPase activity of Vps4p at the yeast multivesicular body. Proc. Natl. Acad. Sci. U.S.A. 103, 6202–6207. 10.1073/pnas.0601712103 16601096 PMC1458855

[B54] Luo Y. Wang Z. Ji H. Fang H. Wang S. Tian L.. (2013). An Arabidopsis homolog of importin β1 is required for ABA response and drought tolerance. Plant J. 75, 377–389. 10.1111/tpj.12207 23582042

[B55] Mahboubi H. Seganathy E. Kong D. Stochaj U. (2013). Identification of novel stress granule components that are involved in nuclear transport. PLoS ONE 8:e68356. 10.1371/journal.pone.0068356 23826389 PMC3694919

[B56] Manders E. M. Stap J. Brakenhoff G. J. van Driel R. Aten J. A. (1992). Dynamics of three-dimensional replication patterns during the S-phase, analysed by double labelling of DNA and confocal microscopy. J. Cell Sci. 103, 857–862. 10.1242/jcs.103.3.857 1478975

[B57] Mathur J. Mathur N. Kirik V. Kernebeck B. Srinivas B. P. Hülskamp M. (2003). Arabidopsis CROOKED encodes for the smallest subunit of the ARP2/3 complex and controls cell shape by region specific fine F-actin formation. Development 130, 3137–3146. 10.1242/dev.00549 12783786

[B58] Mayberry L. K. Leah Allen M. Dennis M. D. Browning K. S. (2009). Evidence for variation in the optimal translation initiation complex: plant eIF4B, eIF4F, and eIF(iso)4F differentially promote translation of mRNAs. Plant Physiol. 150, 1844–1854. 10.1104/pp.109.138438 19493973 PMC2719132

[B59] McCue A. D. Nuthikattu S. Reeder S. H. Slotkin R. K. (2012). Gene expression and stress response mediated by the epigenetic regulation of a transposable element small RNA. PLoS Genet. 8:e1002474. 10.1371/journal.pgen.1002474 22346759 PMC3276544

[B60] Meaden P. G. Arneborg N. Guldfeldt L. U. Siegumfeldt H. Jakobsen M. (1999). Endocytosis and vacuolar morphology in Saccharomyces cerevisiae are altered in response to ethanol stress or heat shock. Yeast 15, 1211–1222. 10.1002/(SICI)1097-0061(19990915)15:12 <1211::AID-YEA448>3.0.CO;2-H 10487923

[B61] Mi H. Dong Q. Muruganujan A. Gaudet P. Lewis S. Thomas P. D. (2009). PANTHER version 7: improved phylogenetic trees, orthologs and collaboration with the Gene Ontology Consortium. Nucleic Acids Res. 38, D204–D210. 10.1093/nar/gkp1019 20015972 PMC2808919

[B62] Murashige T. Skoog F. (1962). A revised medium for rapid growth and bio assays with tobacco tissue cultures. Physiol. Plant. 15, 473–497. 10.1111/j.1399-3054.1962.tb08052.x

[B63] Muthuramalingam M. Wang Y. Li Y. Mahalingam R. (2017). Interacting protein partners of Arabidopsis RNA-binding protein AtRBP45b. Plant Biol. 19, 327–334. 10.1111/plb.12540 28039930

[B64] Nath S. Munsie L. N. Truant R. (2015). A huntingtin-mediated fast stress response halting endosomal trafficking is defective in Huntington's disease. Hum. Mol. Genet. 24, 450–462. 10.1093/hmg/ddu460 25205111 PMC4275073

[B65] Nguyen C. C. Nakaminami K. Matsui A. Kobayashi S. Kurihara Y. Toyooka K.. (2016). Oligouridylate binding protein 1b plays an integral role in plant heat stress tolerance. Front. Plant Sci. 7:853. 10.3389/fpls.2016.00853 27379136 PMC4911357

[B66] Obita T. Saksena S. Ghazi-Tabatabai S. Gill D. J. Perisic O. Emr S. D.. (2007). Structural basis for selective recognition of ESCRT-III by the AAA ATPase Vps4. Nature 449, 735–739. 10.1038/nature06171 17928861

[B67] Olmos Y. Hodgson L. Mantell J. Verkade P. Carlton J. G. (2015). ESCRT-III controls nuclear envelope reformation. Nature 522, 236–239. 10.1038/nature14503 26040713 PMC4471131

[B68] Omer A. Patel D. Lian X. J. Sadek J. Di Marco S. Pause A.. (2018). Stress granules counteract senescence by sequestration of PAI-1. EMBO Rep. 19:e44722. 10.15252/embr.201744722 29592859 PMC5934773

[B69] Parida A. P. Sharma A. Sharma A. K. (2017). AtMBD6, a methyl CpG binding domain protein, maintains gene silencing in Arabidopsis by interacting with RNA binding proteins. J. Biosci. 42, 57–68. 10.1007/s12038-016-9658-1 28229965

[B70] Parker R. Sheth U. (2007). P bodies and the control of mRNA translation and degradation. Mol. Cell 25, 635–646. 10.1016/j.molcel.2007.02.011 17349952

[B71] Pendle A. F. Clark G. P. Boon R. Lewandowska D. Lam Y. W. Andersen J.. (2005). Proteomic analysis of the Arabidopsis nucleolus suggests novel nucleolar functions. Mol. Biol. Cell 16, 260–269. 10.1091/mbc.e04-09-0791 15496452 PMC539170

[B72] Perez-Riverol Y. Csordas A. Bai J. Bernal-Llinares M. Hewapathirana S. Kundu D. J.. (2019). The PRIDE database and related tools and resources in 2019: improving support for quantification data. Nucleic Acids Res. 47, D442–D450. 10.1093/nar/gky1106 30395289 PMC6323896

[B73] Pfaff C. Ehrnsberger H. F. Flores-Tornero M. Sørensen B. B. Schubert T. Längst G.. (2018). ALY RNA-binding proteins are required for nucleocytosolic mRNA transport and modulate plant growth and development. Plant Physiol. 177, 226–240. 10.1104/pp.18.00173 29540591 PMC5933122

[B74] Protter D. S. W. Parker R. (2016). Principles and properties of stress granules. Trends Cell Biol. 26, 668–679. 10.1016/j.tcb.2016.05.004 27289443 PMC4993645

[B75] Pu Y. Bassham D. C. (2016). Detection of autophagy in plants by fluorescence microscopy. Methods Mol. Biol. 1450, 161–172. 10.1007/978-1-4939-3759-2_13 27424753

[B76] Rodrigo-Peiris T. Xu X. M. Zhao Q. Wang H. J. Meier I. (2011). RanGAP is required for post-meiotic mitosis in female gametophyte development in *Arabidopsis thaliana*. J. Exp. Bot. 62, 2705–2714. 10.1093/jxb/erq448 21282324

[B77] Rojo E. Gillmor C. S. Kovaleva V. Somerville C. R. Raikhel N. V. (2001). VACUOLELESS1 IS an essential gene required for vacuole formation and morphogenesis in Arabidopsis. Dev. Cell 1, 303–310. 10.1016/S1534-5807(01)00024-7 11702788

[B78] Rojo E. Zouhar J. Kovaleva V. Hong S. Raikhel N. V. (2003). The AtC-VPS protein complex is localized to the tonoplast and the prevacuolar compartment in arabidopsis. Mol. Biol. Cell 14, 361–369. 10.1091/mbc.e02-08-0509 12589039 PMC149977

[B79] Roy R. Bassham D. C. (2017). TNO1, a TGN-localized SNARE-interacting protein, modulates root skewing in *Arabidopsis thaliana*. BMC Plant Biol. 17, 1–12. 10.1186/s12870-017-1024-4 28399805 PMC5387210

[B80] Saedler R. Jakoby M. Marin B. Galiana-Jaime E. Hülskamp M. (2009). The cell morphogenesis gene SPIRRIG in Arabidopsis encodes a WD/BEACH domain protein. Plant J. 59, 612–621. 10.1111/j.1365-313X.2009.03900.x 19392685

[B81] Scott A. Chung H. Y. Gonciarz-Swiatek M. Hill G. C. Whitby F. G. Gaspar J.. (2005a). Structural and mechanistic studies of VPS4 proteins. EMBO J. 24, 3658–3669. 10.1038/sj.emboj.7600818 16193069 PMC1276703

[B82] Scott A. Gaspar J. Stuchell-Brereton M. D. Alam S. L. Skalicky J. J. Sundquist W. I. (2005b). Structure and ESCRT-III protein interactions of the MIT domain of human VPS4A. Proc. Natl. Acad. Sci. U. S. A. 102, 13813–13818. 10.1073/pnas.0502165102 16174732 PMC1236530

[B83] Sessions A. Burke E. Presting G. Aux G. McElver J. Patton D.. (2002). A high-throughput Arabidopsis reverse genetics system. Plant Cell 14, 2985–2994. 10.1105/tpc.004630 12468722 PMC151197

[B84] Shahriari M. Hülskamp M. Schellmann S. (2010a). Seeds of arabidopsis plants expressing dominant-negative AtSKD1 under control of the GL2 promoter show a transparent testa phenotype and a mucilage defect. Plant Signal. Behav. 5, 1308–1310. 10.4161/psb.5.10.13134 20930567 PMC3115375

[B85] Shahriari M. Keshavaiah C. Scheuring D. Sabovljevic A. Pimpl P. Häusler R. E.. (2010b). The AAA-type ATPase AtSKD1 contributes to vacuolar maintenance of *Arabidopsis thaliana*. Plant J. 64, 71–85. 10.1111/j.1365-313X.2010.04310.x 20663085

[B86] Sohn E. J. Kim E. S. Zhao M. Kim S. J. Kim H. Kim Y. W.. (2003). Rha1, an Arabidopsis Rab5 homolog, plays a critical role in the vacuolar trafficking of soluble cargo proteins. Plant Cell 15, 1057–1070. 10.1105/tpc.009779 12724533 PMC153716

[B87] Sorenson R. Bailey-Serres J. (2014). Selective mRNA sequestration by OLIGOURIDYLATEBINDING PROTEIN 1 contributes to translational control during hypoxia in Arabidopsis. Proc. Natl. Acad. Sci. U.S.A. 111, 2373–2378. 10.1073/pnas.1314851111 24469793 PMC3926019

[B88] Spitzer C. Reyes F. C. Buono R. Sliwinski M. K. Haas T. J. Otegui M. S. (2009). The ESCRT-Related CHMP1A and B proteins mediate multivesicular body sorting of auxin carriers in Arabidopsis and are required for plant development. Plant Cell 21, 749–766. 10.1105/tpc.108.064865 19304934 PMC2671707

[B89] Steffens A. Bräutigam A. Jakoby M. Hülskamp M. Meyer S. (2015). The BEACH domain protein SPIRRIG is essential for arabidopsis salt stress tolerance and functions as a regulator of transcript stabilization and localization. PLoS Biol. 13:e1002188. 10.1371/journal.pbio.1002188 26133670 PMC4489804

[B90] Steffens A. Jakoby M. Hülskamp M. (2017). Physical, functional and genetic interactions between the BEACH domain protein SPIRRIG and LIP5 and SKD1 and its role in endosomal trafficking to the vacuole in Arabidopsis. Front. Plant Sci. 8:1969. 10.3389/fpls.2017.01969 29209342 PMC5701936

[B91] Stephan L. Jakoby M. Hülskamp M. (2021). Evolutionary comparison of the developmental/physiological phenotype and the molecular behavior of spirrig between *Arabidopsis thaliana* and *Arabis alpina*. Front. Plant Sci. 11:2115. 10.3389/fpls.2020.596065 33584744 PMC7874212

[B92] Stuchell-Brereton M. D. Skalicky J. J. Kieffer C. Karren M. A. Ghaffarian S. Sundquist W. I. (2007). ESCRT-III recognition by VPS4 ATPases. Nature 449, 740–744. 10.1038/nature06172 17928862

[B93] Takahara T. Maeda T. (2012). Transient sequestration of TORC1 into stress granules during heat stress. Mol. Cell 47, 242–252. 10.1016/j.molcel.2012.05.019 22727621

[B94] Takemoto K. Ebine K. Askani J. C. Krüger F. Gonzalez Z. A. Ito E.. (2018). Distinct sets of tethering complexes, SNARE complexes, and Rab GTPases mediate membrane fusion at the vacuole in Arabidopsis. Proc. Natl. Acad. Sci. U.S.A. 115, E2457–E2466. 10.1073/pnas.1717839115 29463724 PMC5877921

[B95] Thomas P. D. Campbell M. J. Kejariwal A. Mi H. Karlak B. Daverman R.. (2003). PANTHER: a library of protein families and subfamilies indexed by function. Genome Res. 13, 2129–2141. 10.1101/gr.772403 12952881 PMC403709

[B96] Thompson A. R. Doelling J. H. Suttangkakul A. Vierstra R. D. (2005). Autophagic nutrient recycling in Arabidopsis directed by the ATG8 and ATG12 conjugation pathways. Plant Physiol. 138, 2097–2110. 10.1104/pp.105.060673 16040659 PMC1183398

[B97] Tian Q. Streuli M. Saito H. Schlossman S. F. Anderson P. (1991). A polyadenylate binding protein localized to the granules of cytolytic lymphocytes induces DNA fragmentation in target cells. Cell 67, 629–639. 10.1016/0092-8674(91)90536-8 1934064

[B98] Ueda T. Yamaguchi M. Uchimiya H. Nakano A. (2001). Ara6, a plant-unique novel type Rab GTPase, functions in the endocytic pathway of *Arabidopsis thaliana*. EMBO J. 20, 4730–4741. 10.1093/emboj/20.17.4730 11532937 PMC125591

[B99] Vajjhala P. R. Wong J. S. To H. Y. Munn A. L. (2006). The β domain is required for Vps4p oligomerization into a functionally active ATPase. FEBS J. 273, 2357–2373. 10.1111/j.1742-4658.2006.05238.x 16704411

[B100] Vietri M. Schink K. O. Campsteijn C. Wegner C. S. Schultz S. W. Christ L.. (2015). Spastin and ESCRT-III coordinate mitotic spindle disassembly and nuclear envelope sealing. Nature 522, 231–235. 10.1038/nature14408 26040712

[B101] Waidmann S. Kusenda B. Mayerhofer J. Mechtler K. Jonak C. (2014). A dek Domain-containing protein modulates chromatin structure and function in arabidopsisw open. Plant Cell 26, 4328–4344. 10.1105/tpc.114.129254 25387881 PMC4277211

[B102] Wang H.-J. Hsu Y.-W. Guo C.-L. Jane W.-N. Wang H. Jiang L.. (2017). VPS36-Dependent multivesicular bodies are critical for plasmamembrane protein turnover and vacuolar biogenesis 1[OPEN]. Plant Physiol. Ò 173, 566–581. 10.1104/pp.16.01356 PMC5210736 27879389

[B103] Wang M. Li X. Luo S. Fan B. Zhu C. Chen Z. (2020). Coordination and crosstalk between autophagosome and multivesicular body pathways in plant stress responses. Cells 9:119. 10.3390/cells9010119 31947769 PMC7017292

[B104] Weber C. Nover L. Fauth M. (2008). Plant stress granules and mRNA processing bodies are distinct from heat stress granules. Plant J. 56, 517–530. 10.1111/j.1365-313X.2008.03623.x 18643965

[B105] Wippich F. Bodenmiller B. Trajkovska M. G. Wanka S. Aebersold R. Pelkmans L. (2013). Dual specificity kinase DYRK3 couples stress granule condensation/ dissolution to mTORC1 signaling. Cell 152, 791–805. 10.1016/j.cell.2013.01.033 23415227

[B106] Xiao J. Xia H. Yoshino-Koh K. Zhou J. Xu Z. (2007). structural characterization of the ATPase reaction cycle of endosomal AAA Protein Vps4. J. Mol. Biol. 374, 655–670. 10.1016/j.jmb.2007.09.067 17949747 PMC2693005

[B107] Xie X. Matsumoto S. Endo A. Fukushima T. Kawahara H. Saeki Y.. (2018). Deubiquitylases USP5 and USP13 are recruited to and regulate heat-induced stress granules through their deubiquitylating activities. J. Cell Sci. 131:jcs210856. 10.1242/jcs.210856 29567855

[B108] Xu J. Chua N.-H. (2009). Arabidopsis decapping 5 is required for mRNA decapping, P-body formation, and translational repression during postembryonic development. Plant Cell 21, 3270–3279. 10.1105/tpc.109.070078 19855049 PMC2782270

[B109] Xu J. Yang J. Y. Niu Q. W. Chua N. H. (2006). Arabidopsis DCP2, DCP1, and VARICOSE form a decapping complex required for postembryonic development. Plant Cell 18, 3386–3398. 10.1105/tpc.106.047605 17158604 PMC1785416

[B110] Yoshimoto K. Jikumaru Y. Kamiya Y. Kusano M. Consonni C. Panstruga R.. (2009). Autophagy negatively regulates cell death by controlling NPR1-dependent salicylic acid signaling during senescence and the innate immune response in arabidopsis. Plant Cell 21, 2914–2927. 10.1105/tpc.109.068635 19773385 PMC2768913

[B111] Zhuang X. Cui Y. Gao C. Jiang L. (2015). Endocytic and autophagic pathways crosstalk in plants. Curr. Opin. Plant Biol. 28, 39–47. 10.1016/j.pbi.2015.08.010 26453966

