# Supplementary material for: Heat Stress-Dependent Association of Membrane Trafficking Proteins With mRNPs Is Selective
Source: Front Plant Sci. 2021 Jun 24;12:670499. doi: 10.3389/fpls.2021.670499 (PMC8264791; doi:10.3389/fpls.2021.670499)
Supplement: Supplementary Figure 1 — FM4-64 staining of 35S::GFP-SKD1 roots. 35S::GFP-SKD1 roots of 5 or 7d old seedlings were stained with 50 μM FM4-64 in 12 MS liquid medium for 5 min. Cells were incubated at room temperature for 2 h and then subjected to heat treatment. Roots were analyzed by CLSM after heat treatment and single planes of root epidermal cells of the proximal transformation zone/early elongation zone are depicted. Scale bar = 20 μm. [file Presentation_1.PPTX]

## Slide 1
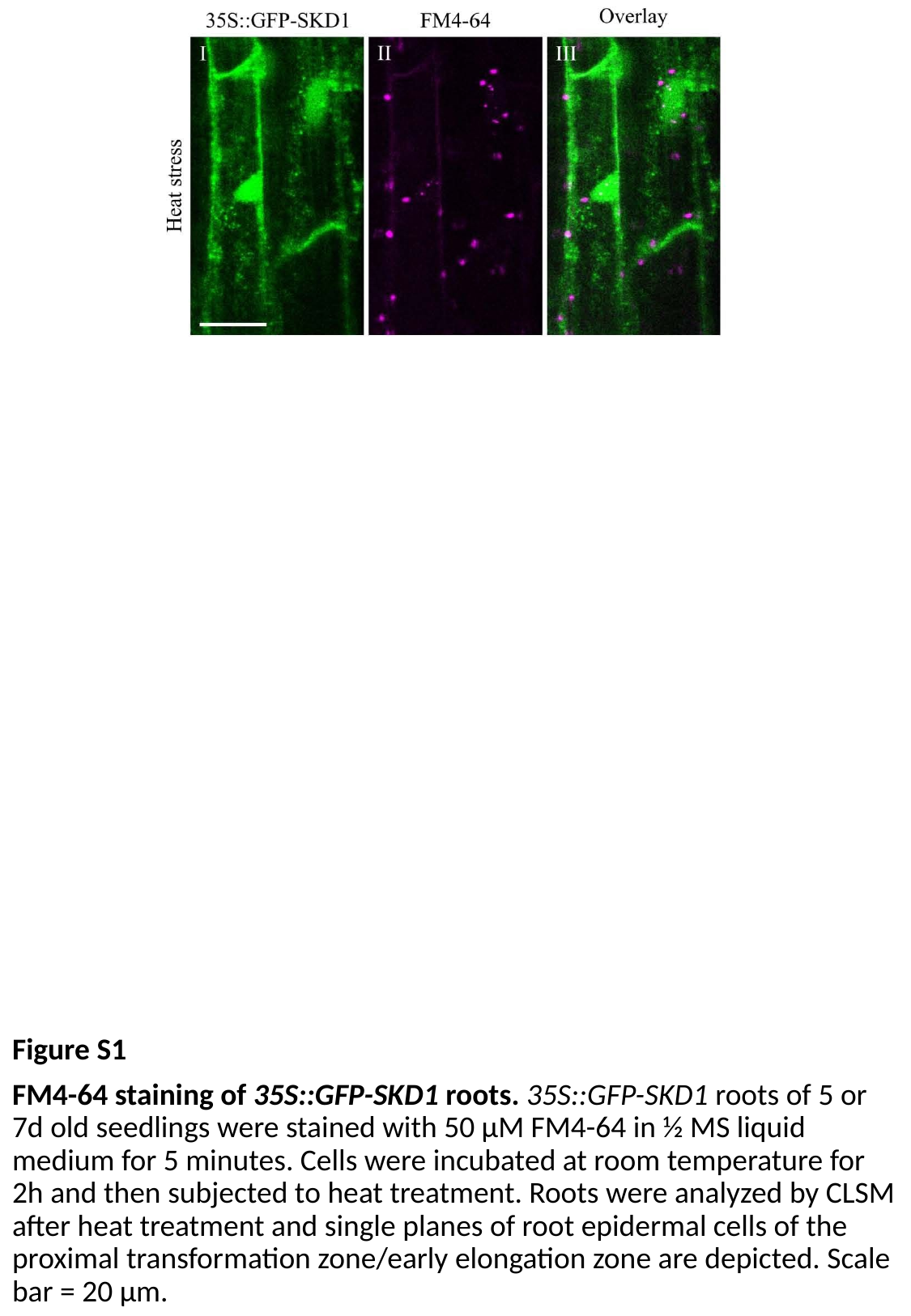

Figure S1
FM4-64 staining of 35S::GFP-SKD1 roots. 35S::GFP-SKD1 roots of 5 or 7d old seedlings were stained with 50 µM FM4-64 in ½ MS liquid medium for 5 minutes. Cells were incubated at room temperature for 2h and then subjected to heat treatment. Roots were analyzed by CLSM after heat treatment and single planes of root epidermal cells of the proximal transformation zone/early elongation zone are depicted. Scale bar = 20 µm.

## Slide 2
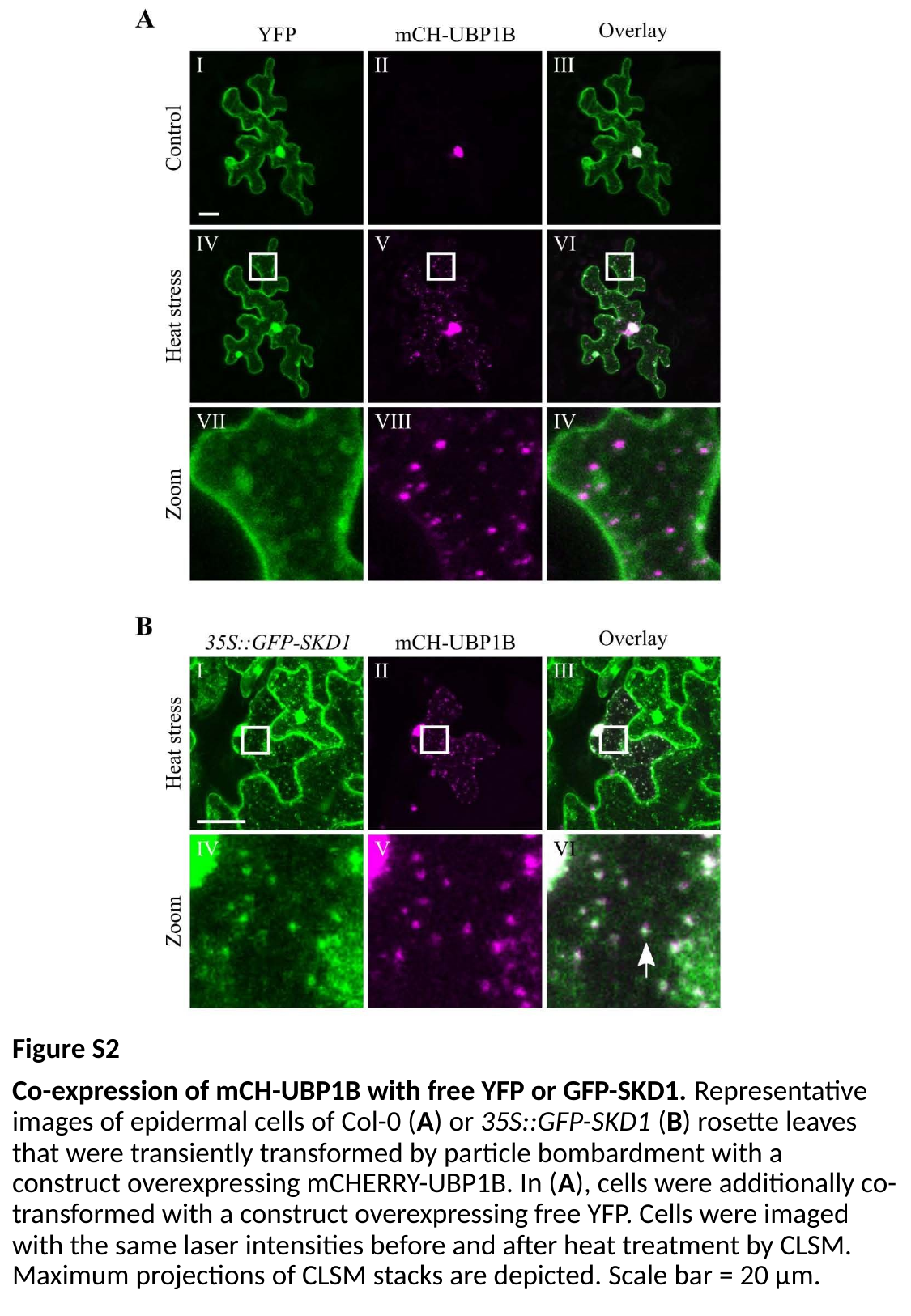

Figure S2
Co-expression of mCH-UBP1B with free YFP or GFP-SKD1. Representative images of epidermal cells of Col-0 (A) or 35S::GFP-SKD1 (B) rosette leaves that were transiently transformed by particle bombardment with a construct overexpressing mCHERRY-UBP1B. In (A), cells were additionally co-transformed with a construct overexpressing free YFP. Cells were imaged with the same laser intensities before and after heat treatment by CLSM. Maximum projections of CLSM stacks are depicted. Scale bar = 20 µm.

## Slide 3
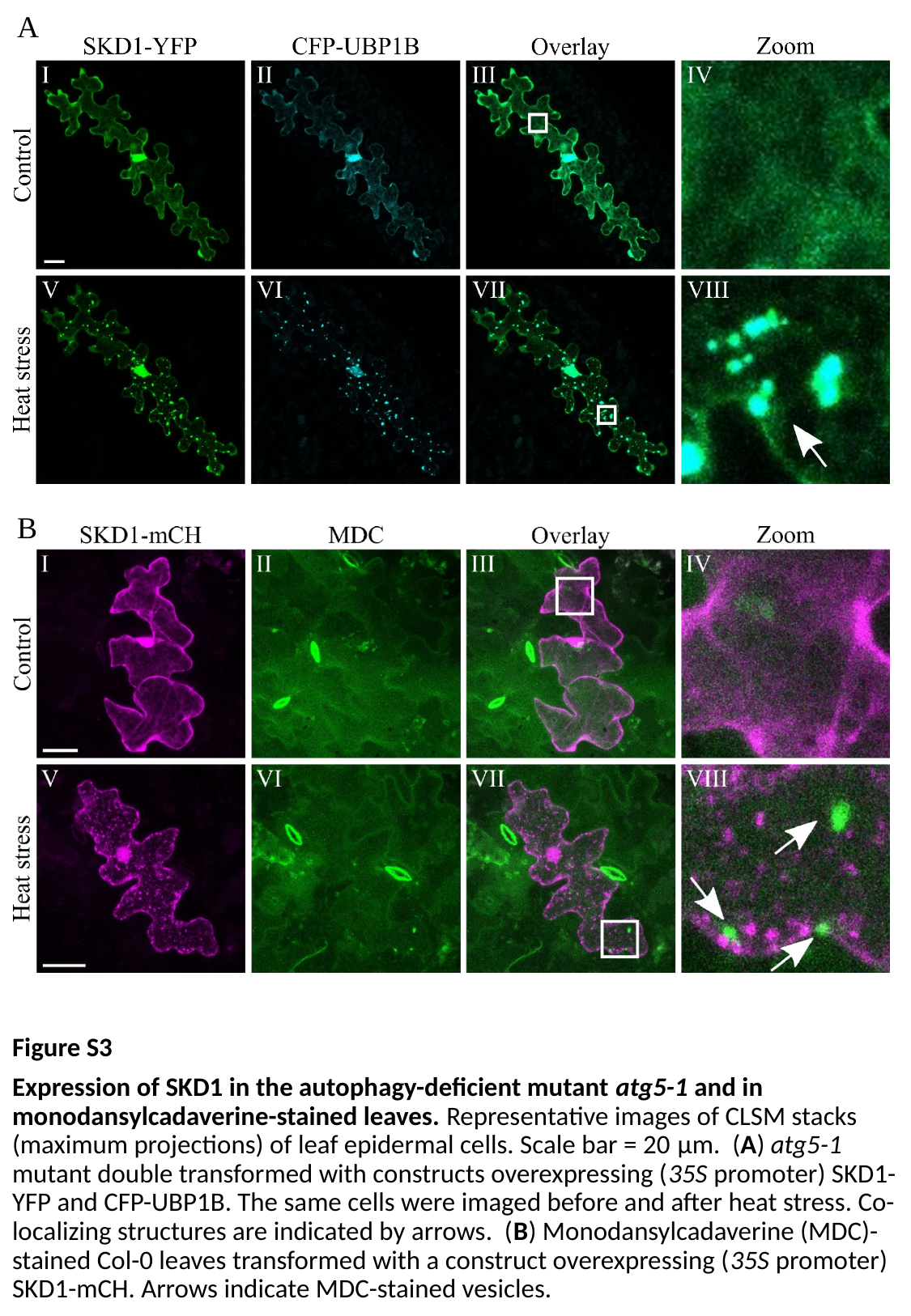

A
B
Figure S3
Expression of SKD1 in the autophagy-deficient mutant atg5-1 and in monodansylcadaverine-stained leaves. Representative images of CLSM stacks (maximum projections) of leaf epidermal cells. Scale bar = 20 µm. (A) atg5-1 mutant double transformed with constructs overexpressing (35S promoter) SKD1-YFP and CFP-UBP1B. The same cells were imaged before and after heat stress. Co-localizing structures are indicated by arrows. (B) Monodansylcadaverine (MDC)-stained Col-0 leaves transformed with a construct overexpressing (35S promoter) SKD1-mCH. Arrows indicate MDC-stained vesicles.

## Slide 4
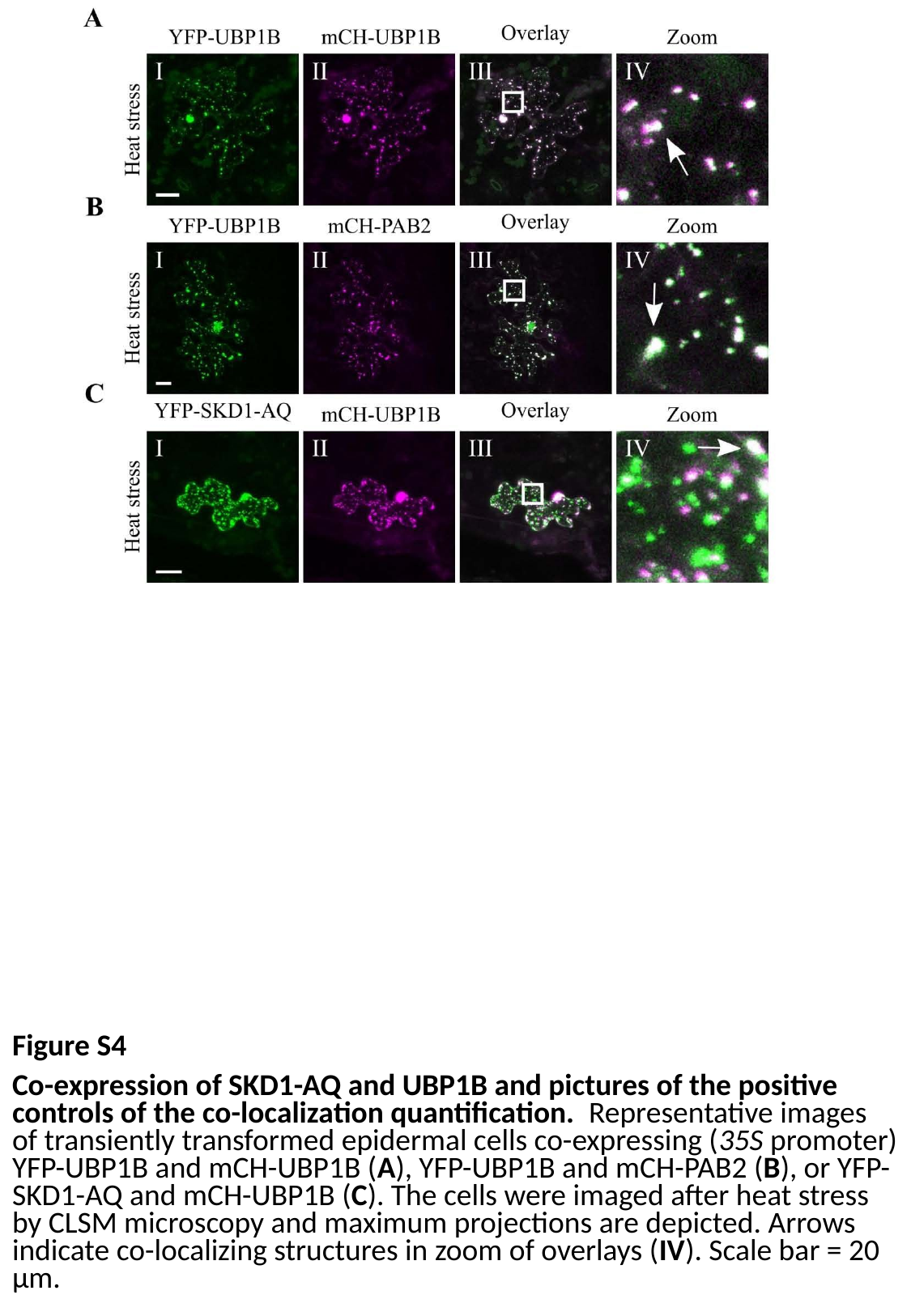

#
Figure S4
Co-expression of SKD1-AQ and UBP1B and pictures of the positive controls of the co-localization quantification.  Representative images of transiently transformed epidermal cells co-expressing (35S promoter) YFP-UBP1B and mCH-UBP1B (A), YFP-UBP1B and mCH-PAB2 (B), or YFP-SKD1-AQ and mCH-UBP1B (C). The cells were imaged after heat stress by CLSM microscopy and maximum projections are depicted. Arrows indicate co-localizing structures in zoom of overlays (IV). Scale bar = 20 µm.

## Slide 5
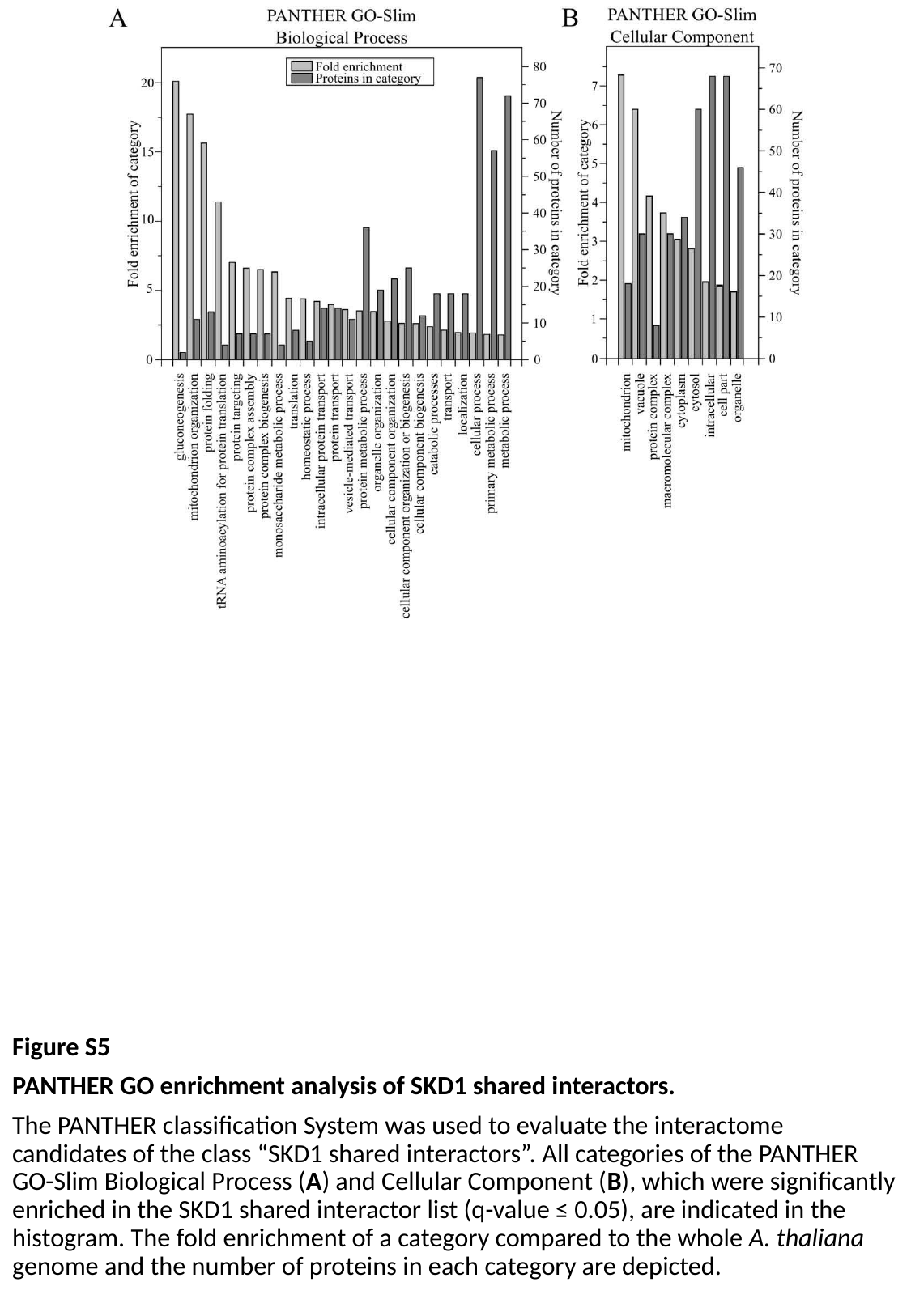

#
Figure S5
PANTHER GO enrichment analysis of SKD1 shared interactors.
The PANTHER classification System was used to evaluate the interactome candidates of the class “SKD1 shared interactors”. All categories of the PANTHER GO-Slim Biological Process (A) and Cellular Component (B), which were significantly enriched in the SKD1 shared interactor list (q-value ≤ 0.05), are indicated in the histogram. The fold enrichment of a category compared to the whole A. thaliana genome and the number of proteins in each category are depicted.

## Slide 6
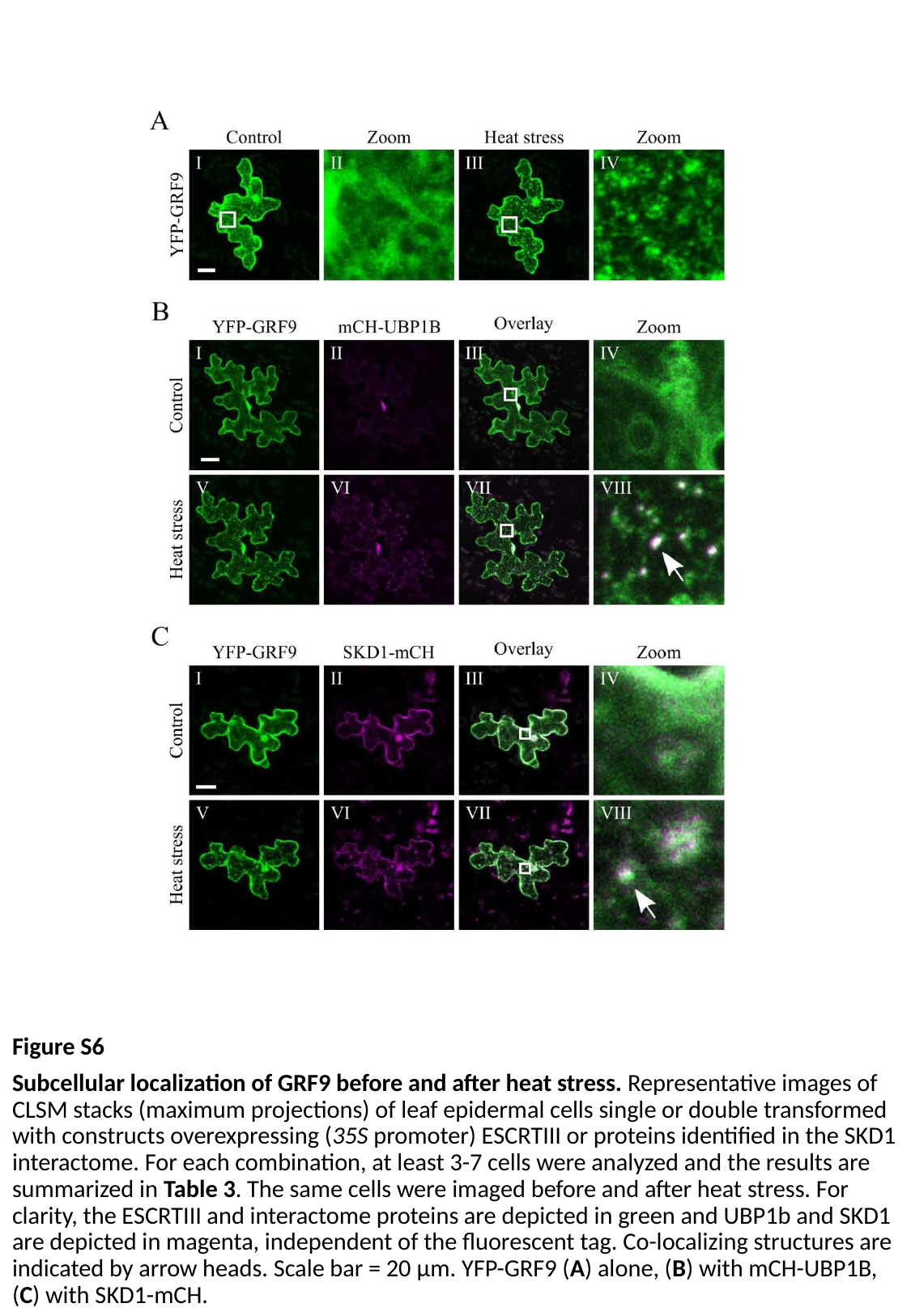

Figure S6
Subcellular localization of GRF9 before and after heat stress. Representative images of CLSM stacks (maximum projections) of leaf epidermal cells single or double transformed with constructs overexpressing (35S promoter) ESCRTIII or proteins identified in the SKD1 interactome. For each combination, at least 3-7 cells were analyzed and the results are summarized in Table 3. The same cells were imaged before and after heat stress. For clarity, the ESCRTIII and interactome proteins are depicted in green and UBP1b and SKD1 are depicted in magenta, independent of the fluorescent tag. Co-localizing structures are indicated by arrow heads. Scale bar = 20 µm. YFP-GRF9 (A) alone, (B) with mCH-UBP1B, (C) with SKD1-mCH.

## Slide 7
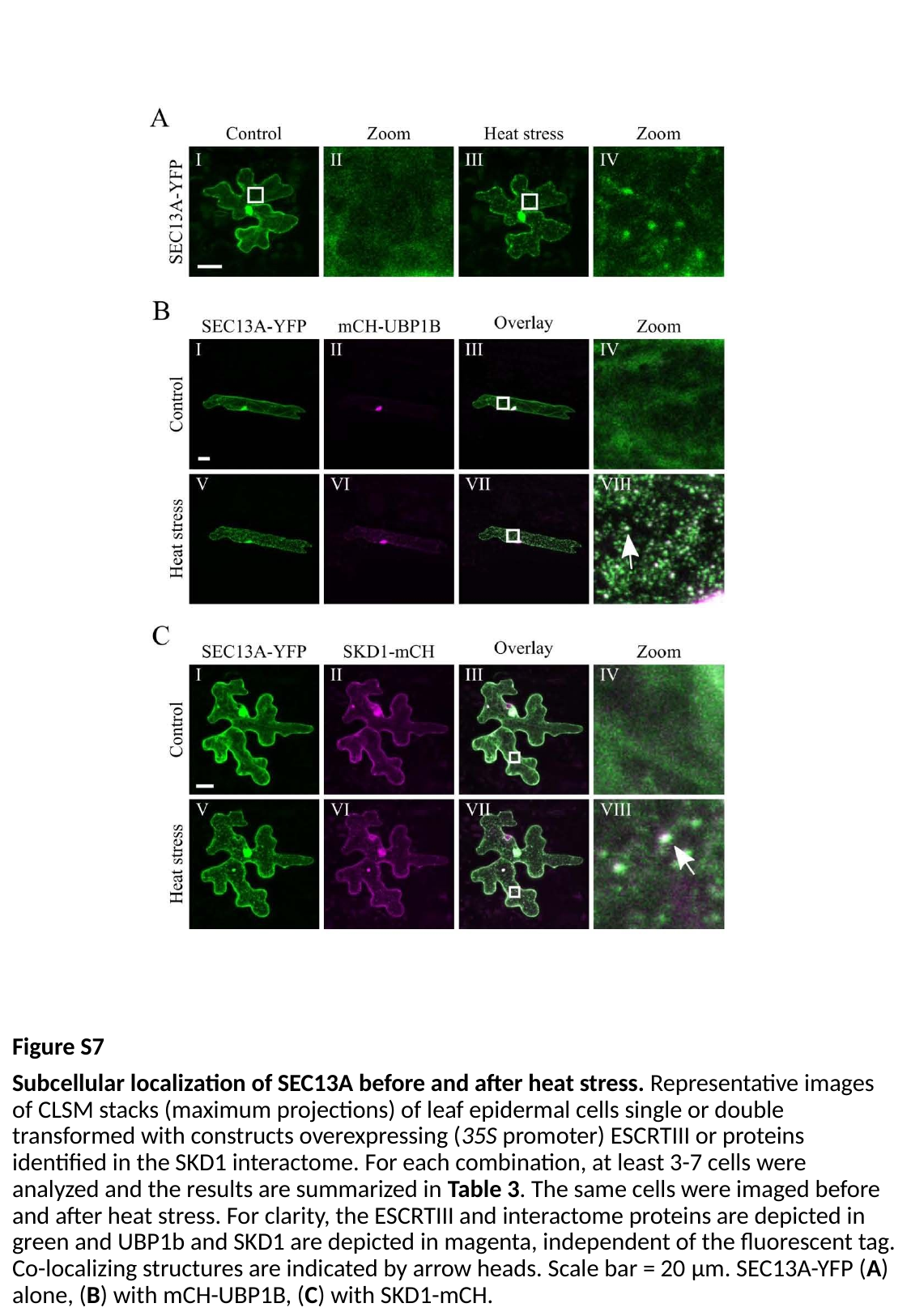

#
Figure S7
Subcellular localization of SEC13A before and after heat stress. Representative images of CLSM stacks (maximum projections) of leaf epidermal cells single or double transformed with constructs overexpressing (35S promoter) ESCRTIII or proteins identified in the SKD1 interactome. For each combination, at least 3-7 cells were analyzed and the results are summarized in Table 3. The same cells were imaged before and after heat stress. For clarity, the ESCRTIII and interactome proteins are depicted in green and UBP1b and SKD1 are depicted in magenta, independent of the fluorescent tag. Co-localizing structures are indicated by arrow heads. Scale bar = 20 µm. SEC13A-YFP (A) alone, (B) with mCH-UBP1B, (C) with SKD1-mCH.

## Slide 8
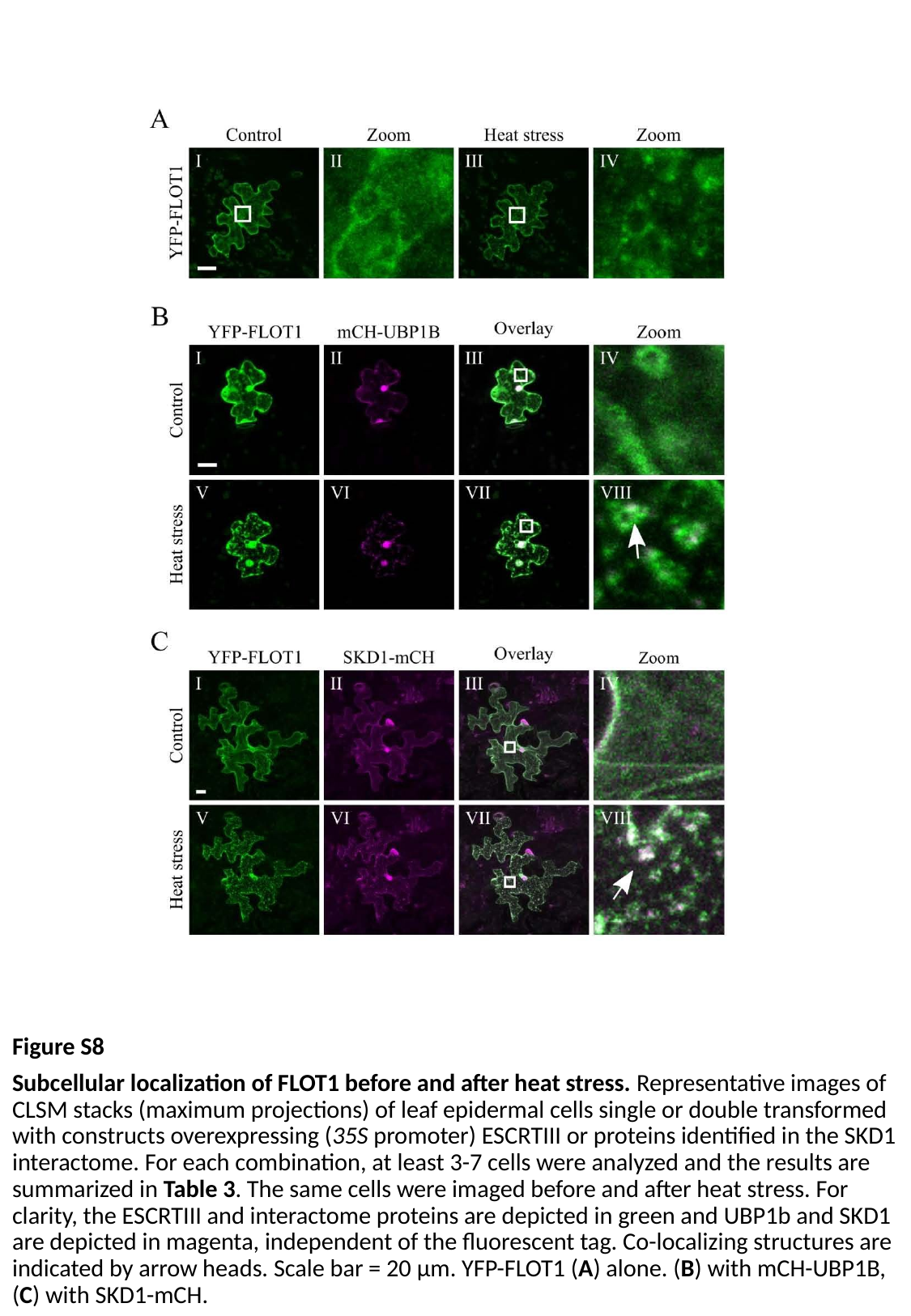

#
Figure S8
Subcellular localization of FLOT1 before and after heat stress. Representative images of CLSM stacks (maximum projections) of leaf epidermal cells single or double transformed with constructs overexpressing (35S promoter) ESCRTIII or proteins identified in the SKD1 interactome. For each combination, at least 3-7 cells were analyzed and the results are summarized in Table 3. The same cells were imaged before and after heat stress. For clarity, the ESCRTIII and interactome proteins are depicted in green and UBP1b and SKD1 are depicted in magenta, independent of the fluorescent tag. Co-localizing structures are indicated by arrow heads. Scale bar = 20 µm. YFP-FLOT1 (A) alone. (B) with mCH-UBP1B, (C) with SKD1-mCH.

## Slide 9
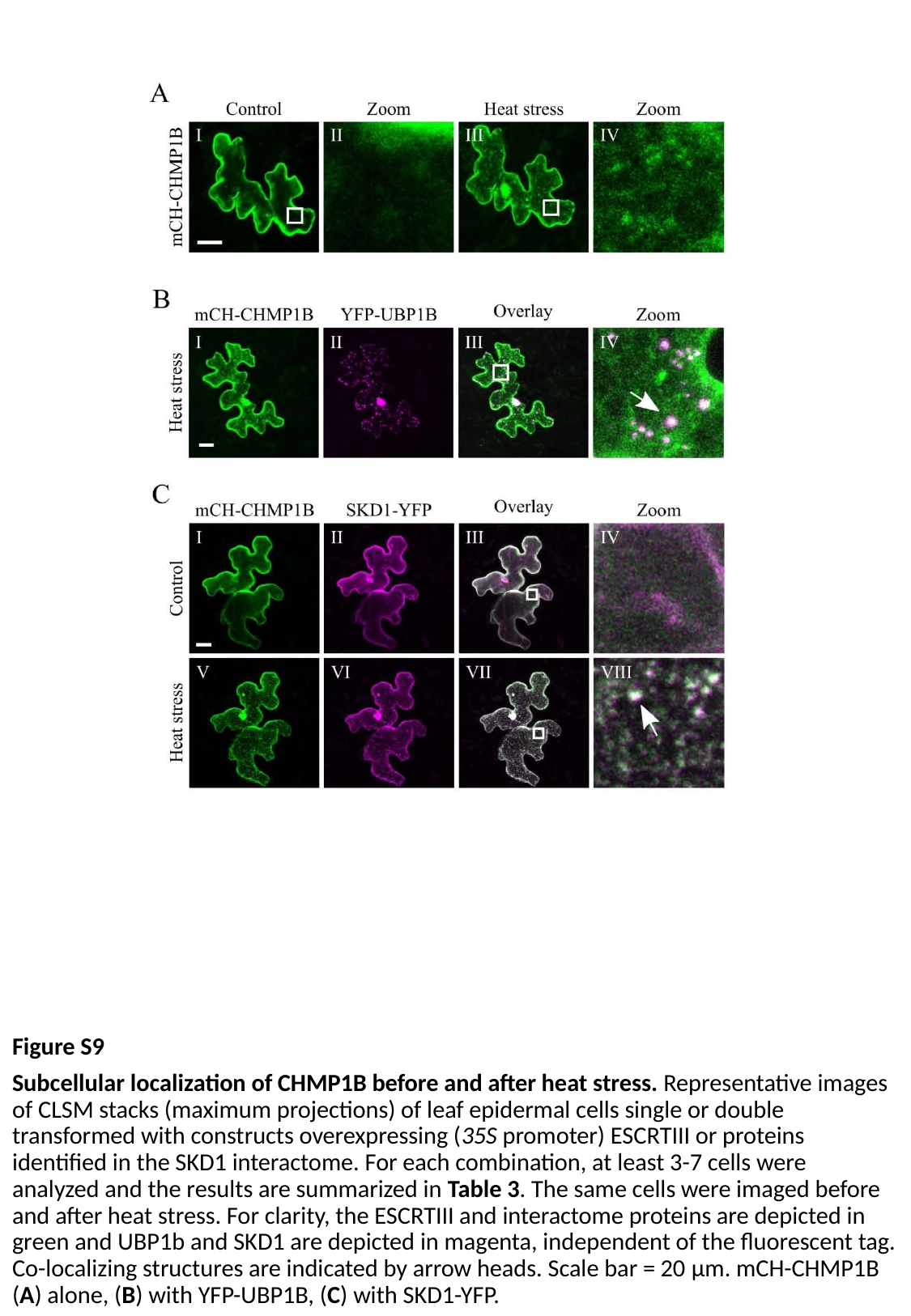

#
Figure S9
Subcellular localization of CHMP1B before and after heat stress. Representative images of CLSM stacks (maximum projections) of leaf epidermal cells single or double transformed with constructs overexpressing (35S promoter) ESCRTIII or proteins identified in the SKD1 interactome. For each combination, at least 3-7 cells were analyzed and the results are summarized in Table 3. The same cells were imaged before and after heat stress. For clarity, the ESCRTIII and interactome proteins are depicted in green and UBP1b and SKD1 are depicted in magenta, independent of the fluorescent tag. Co-localizing structures are indicated by arrow heads. Scale bar = 20 µm. mCH-CHMP1B (A) alone, (B) with YFP-UBP1B, (C) with SKD1-YFP.

## Slide 10
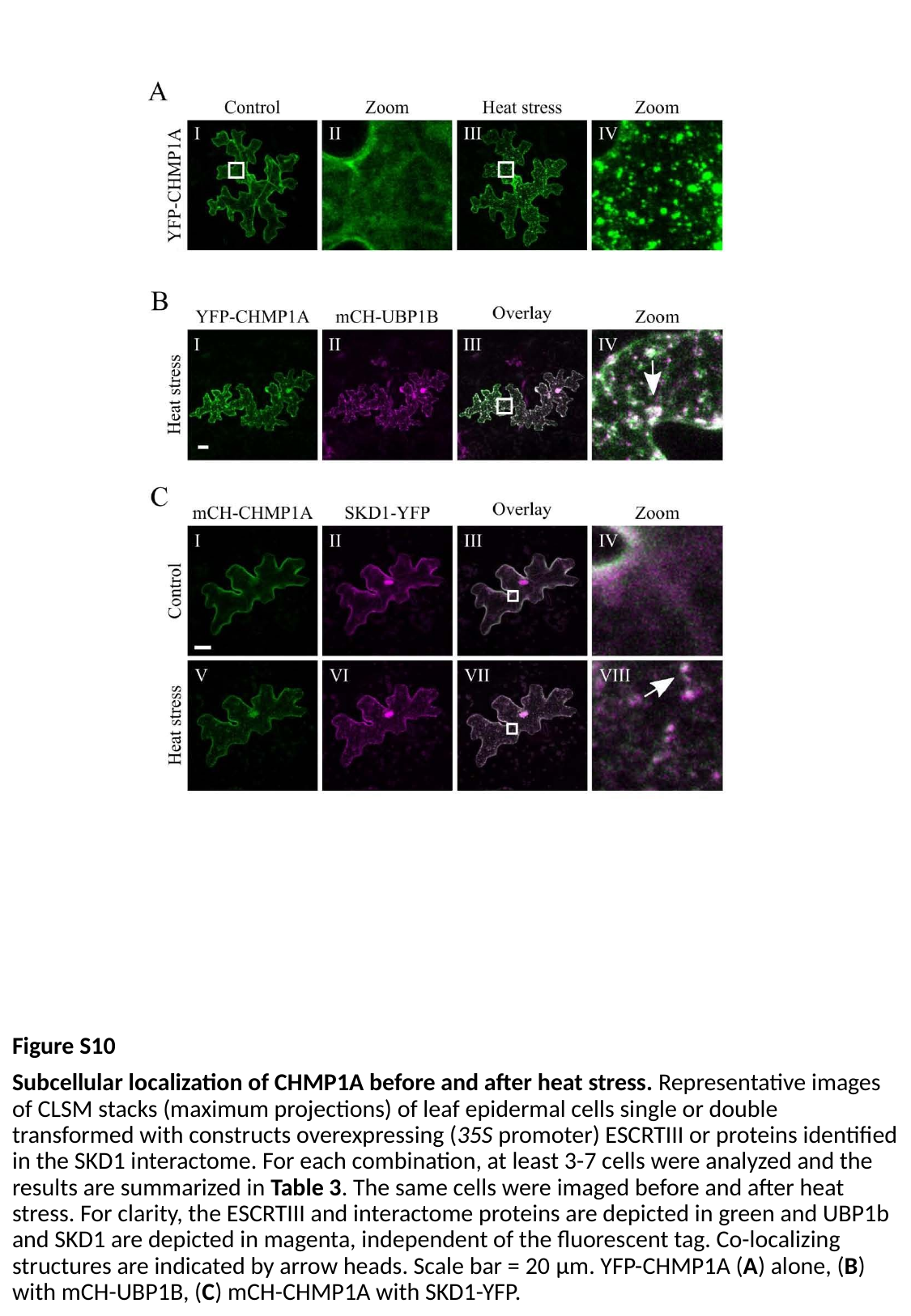

#
Figure S10
Subcellular localization of CHMP1A before and after heat stress. Representative images of CLSM stacks (maximum projections) of leaf epidermal cells single or double transformed with constructs overexpressing (35S promoter) ESCRTIII or proteins identified in the SKD1 interactome. For each combination, at least 3-7 cells were analyzed and the results are summarized in Table 3. The same cells were imaged before and after heat stress. For clarity, the ESCRTIII and interactome proteins are depicted in green and UBP1b and SKD1 are depicted in magenta, independent of the fluorescent tag. Co-localizing structures are indicated by arrow heads. Scale bar = 20 µm. YFP-CHMP1A (A) alone, (B) with mCH-UBP1B, (C) mCH-CHMP1A with SKD1-YFP.

## Slide 11
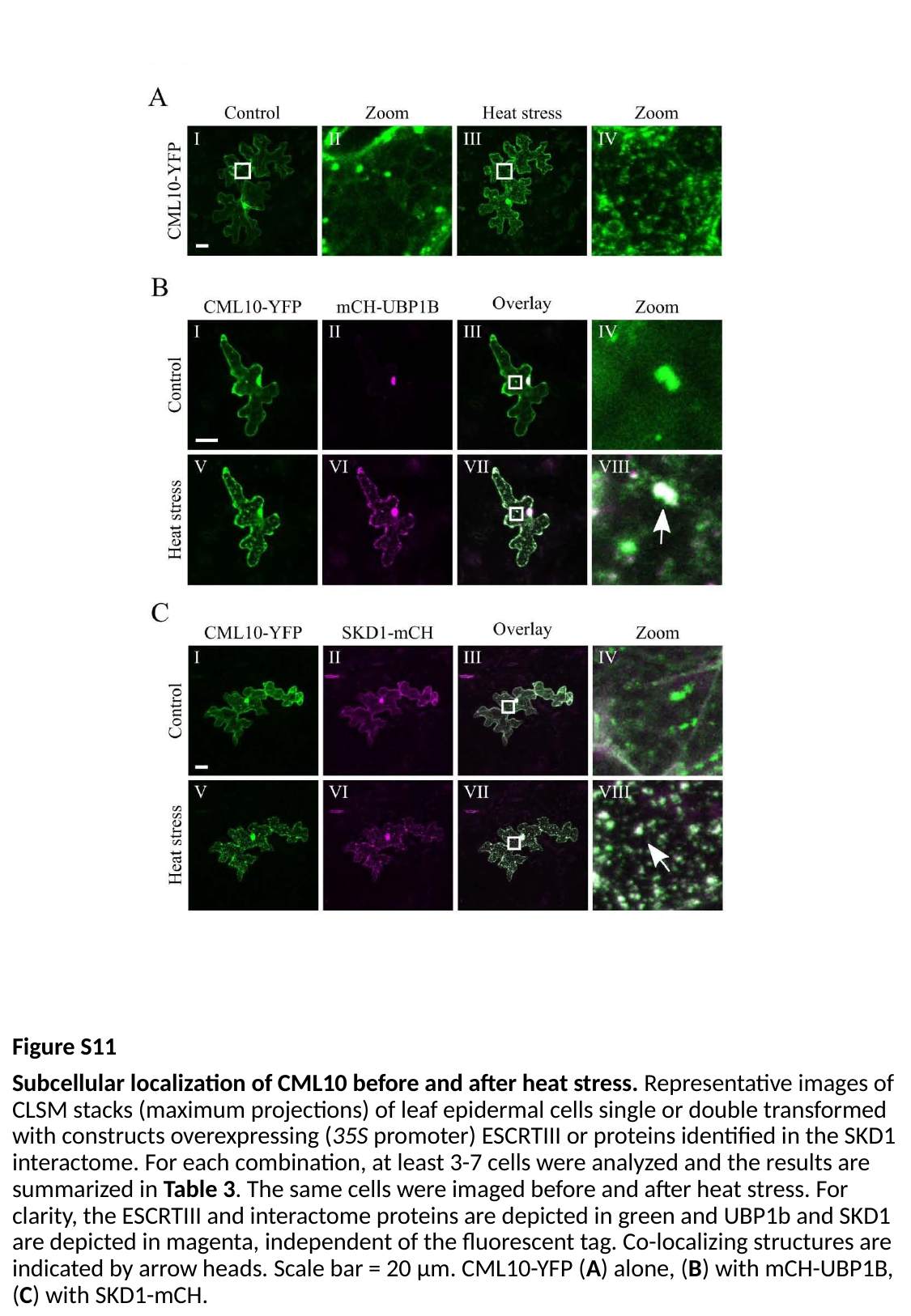

#
Figure S11
Subcellular localization of CML10 before and after heat stress. Representative images of CLSM stacks (maximum projections) of leaf epidermal cells single or double transformed with constructs overexpressing (35S promoter) ESCRTIII or proteins identified in the SKD1 interactome. For each combination, at least 3-7 cells were analyzed and the results are summarized in Table 3. The same cells were imaged before and after heat stress. For clarity, the ESCRTIII and interactome proteins are depicted in green and UBP1b and SKD1 are depicted in magenta, independent of the fluorescent tag. Co-localizing structures are indicated by arrow heads. Scale bar = 20 µm. CML10-YFP (A) alone, (B) with mCH-UBP1B, (C) with SKD1-mCH.

## Slide 12
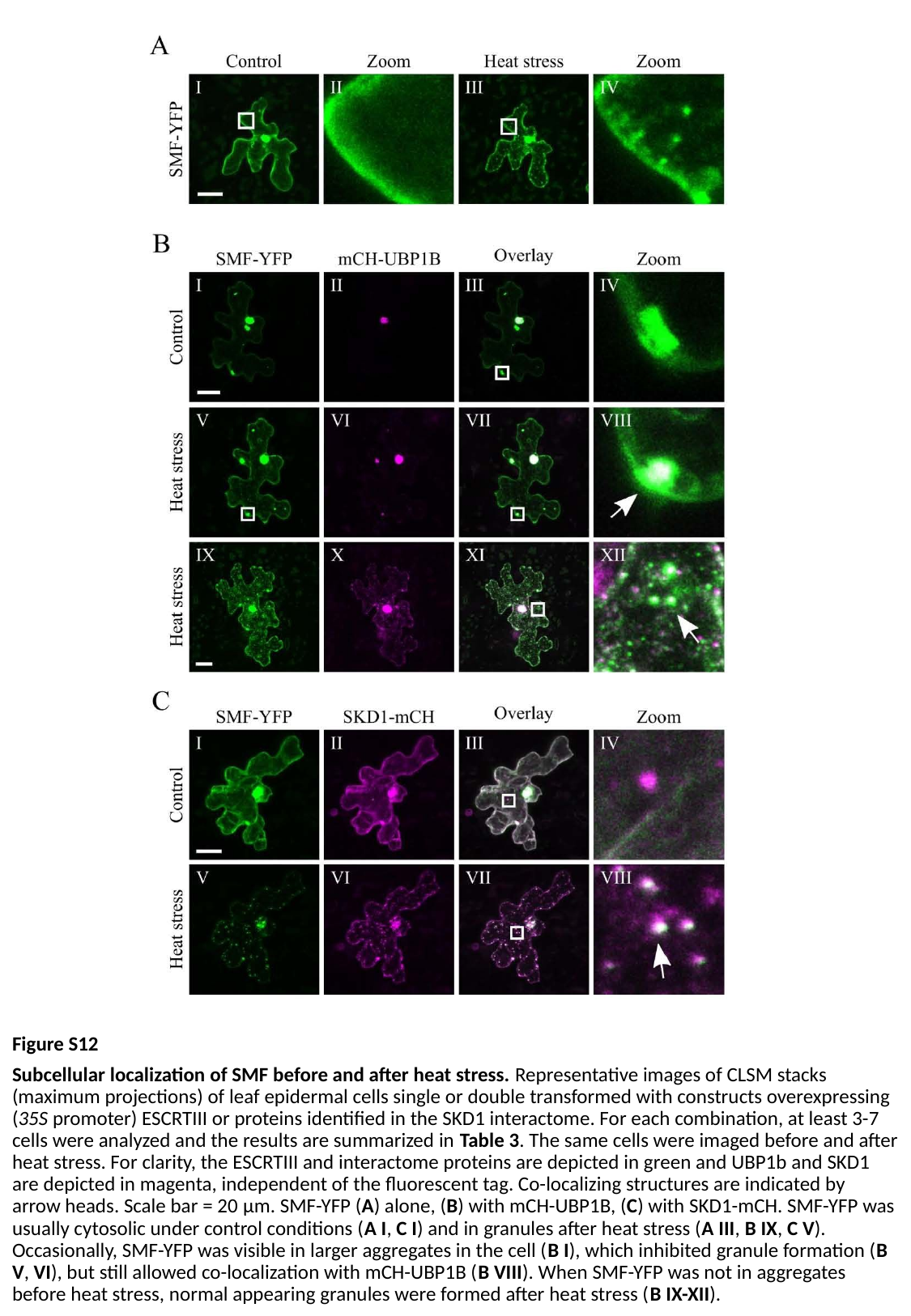

#
Figure S12
Subcellular localization of SMF before and after heat stress. Representative images of CLSM stacks (maximum projections) of leaf epidermal cells single or double transformed with constructs overexpressing (35S promoter) ESCRTIII or proteins identified in the SKD1 interactome. For each combination, at least 3-7 cells were analyzed and the results are summarized in Table 3. The same cells were imaged before and after heat stress. For clarity, the ESCRTIII and interactome proteins are depicted in green and UBP1b and SKD1 are depicted in magenta, independent of the fluorescent tag. Co-localizing structures are indicated by arrow heads. Scale bar = 20 µm. SMF-YFP (A) alone, (B) with mCH-UBP1B, (C) with SKD1-mCH. SMF-YFP was usually cytosolic under control conditions (A I, C I) and in granules after heat stress (A III, B IX, C V). Occasionally, SMF-YFP was visible in larger aggregates in the cell (B I), which inhibited granule formation (B V, VI), but still allowed co-localization with mCH-UBP1B (B VIII). When SMF-YFP was not in aggregates before heat stress, normal appearing granules were formed after heat stress (B IX-XII).

## Slide 13
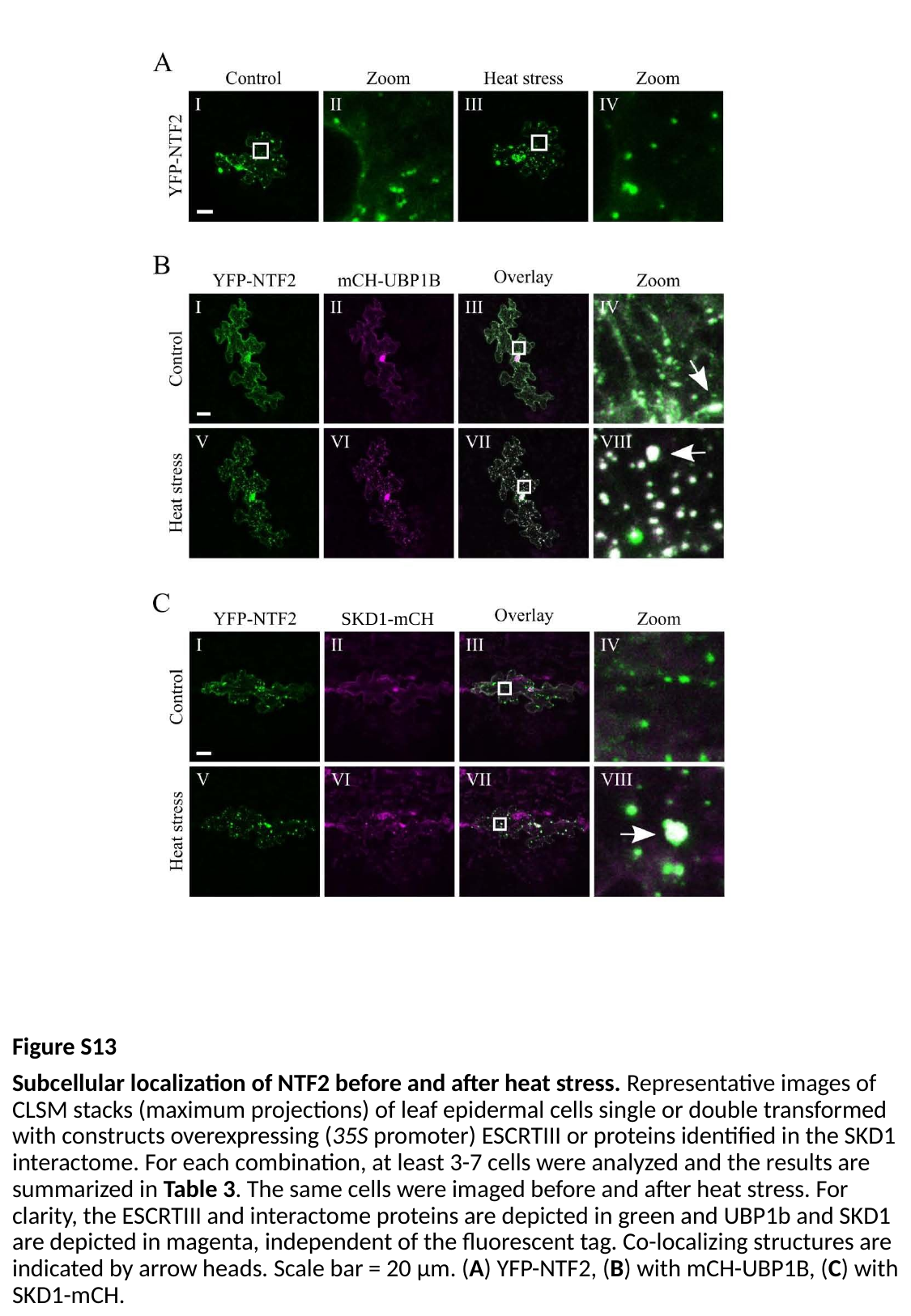

SKD1-mCH
Figure S13
Subcellular localization of NTF2 before and after heat stress. Representative images of CLSM stacks (maximum projections) of leaf epidermal cells single or double transformed with constructs overexpressing (35S promoter) ESCRTIII or proteins identified in the SKD1 interactome. For each combination, at least 3-7 cells were analyzed and the results are summarized in Table 3. The same cells were imaged before and after heat stress. For clarity, the ESCRTIII and interactome proteins are depicted in green and UBP1b and SKD1 are depicted in magenta, independent of the fluorescent tag. Co-localizing structures are indicated by arrow heads. Scale bar = 20 µm. (A) YFP-NTF2, (B) with mCH-UBP1B, (C) with SKD1-mCH.

## Slide 14
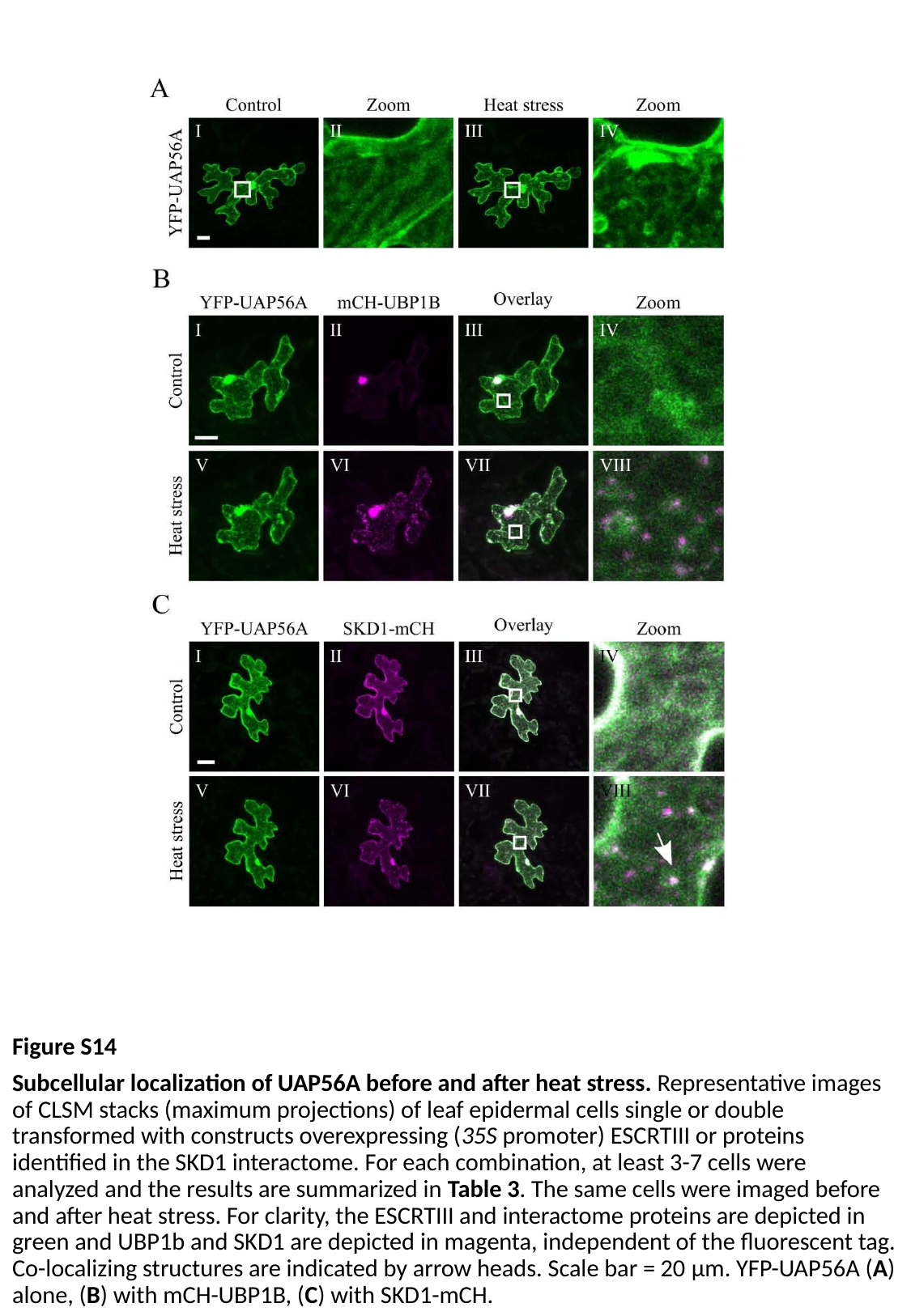

Figure S14
Subcellular localization of UAP56A before and after heat stress. Representative images of CLSM stacks (maximum projections) of leaf epidermal cells single or double transformed with constructs overexpressing (35S promoter) ESCRTIII or proteins identified in the SKD1 interactome. For each combination, at least 3-7 cells were analyzed and the results are summarized in Table 3. The same cells were imaged before and after heat stress. For clarity, the ESCRTIII and interactome proteins are depicted in green and UBP1b and SKD1 are depicted in magenta, independent of the fluorescent tag. Co-localizing structures are indicated by arrow heads. Scale bar = 20 µm. YFP-UAP56A (A) alone, (B) with mCH-UBP1B, (C) with SKD1-mCH.

## Slide 15
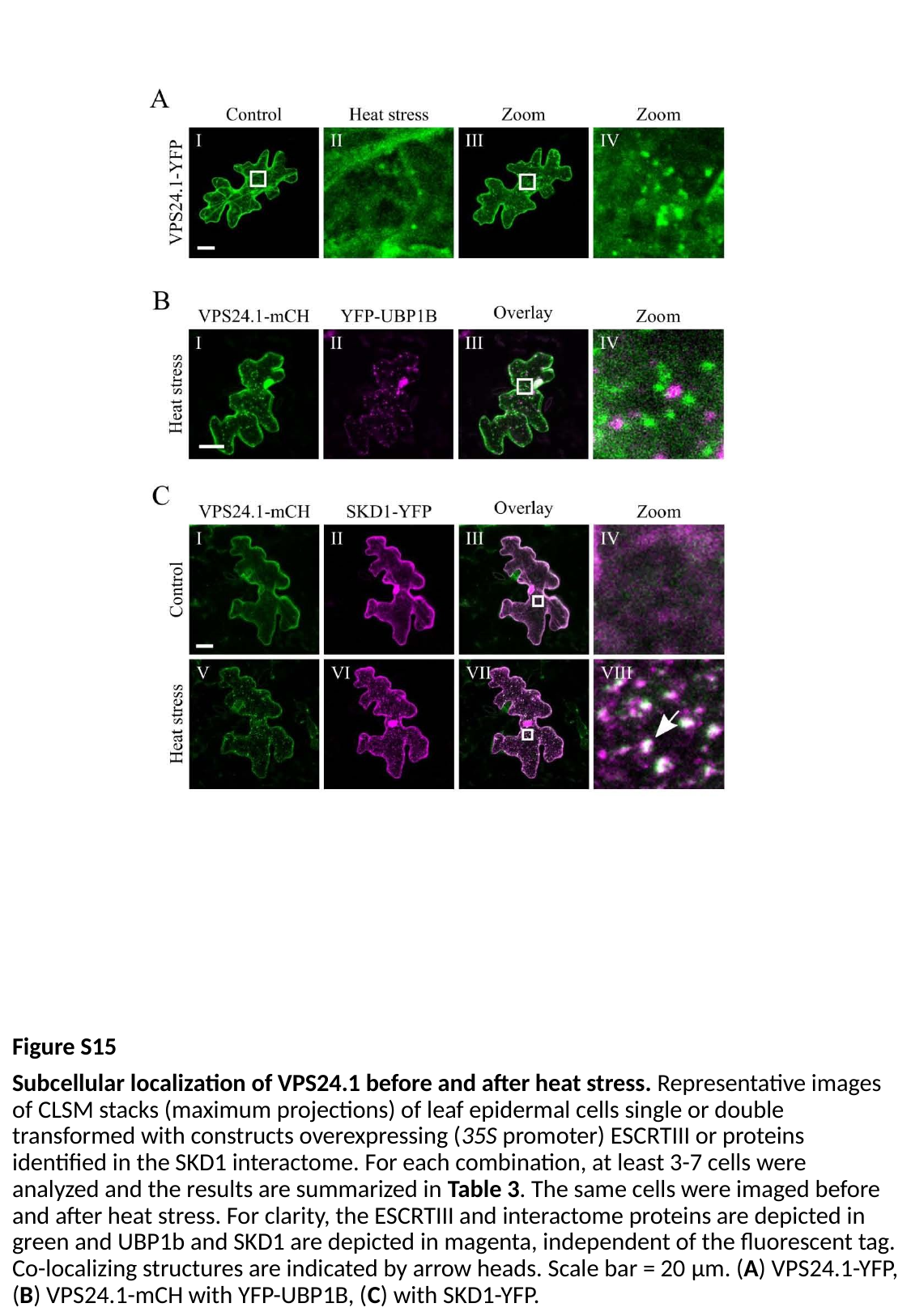

#
Figure S15
Subcellular localization of VPS24.1 before and after heat stress. Representative images of CLSM stacks (maximum projections) of leaf epidermal cells single or double transformed with constructs overexpressing (35S promoter) ESCRTIII or proteins identified in the SKD1 interactome. For each combination, at least 3-7 cells were analyzed and the results are summarized in Table 3. The same cells were imaged before and after heat stress. For clarity, the ESCRTIII and interactome proteins are depicted in green and UBP1b and SKD1 are depicted in magenta, independent of the fluorescent tag. Co-localizing structures are indicated by arrow heads. Scale bar = 20 µm. (A) VPS24.1-YFP, (B) VPS24.1-mCH with YFP-UBP1B, (C) with SKD1-YFP.

## Slide 16
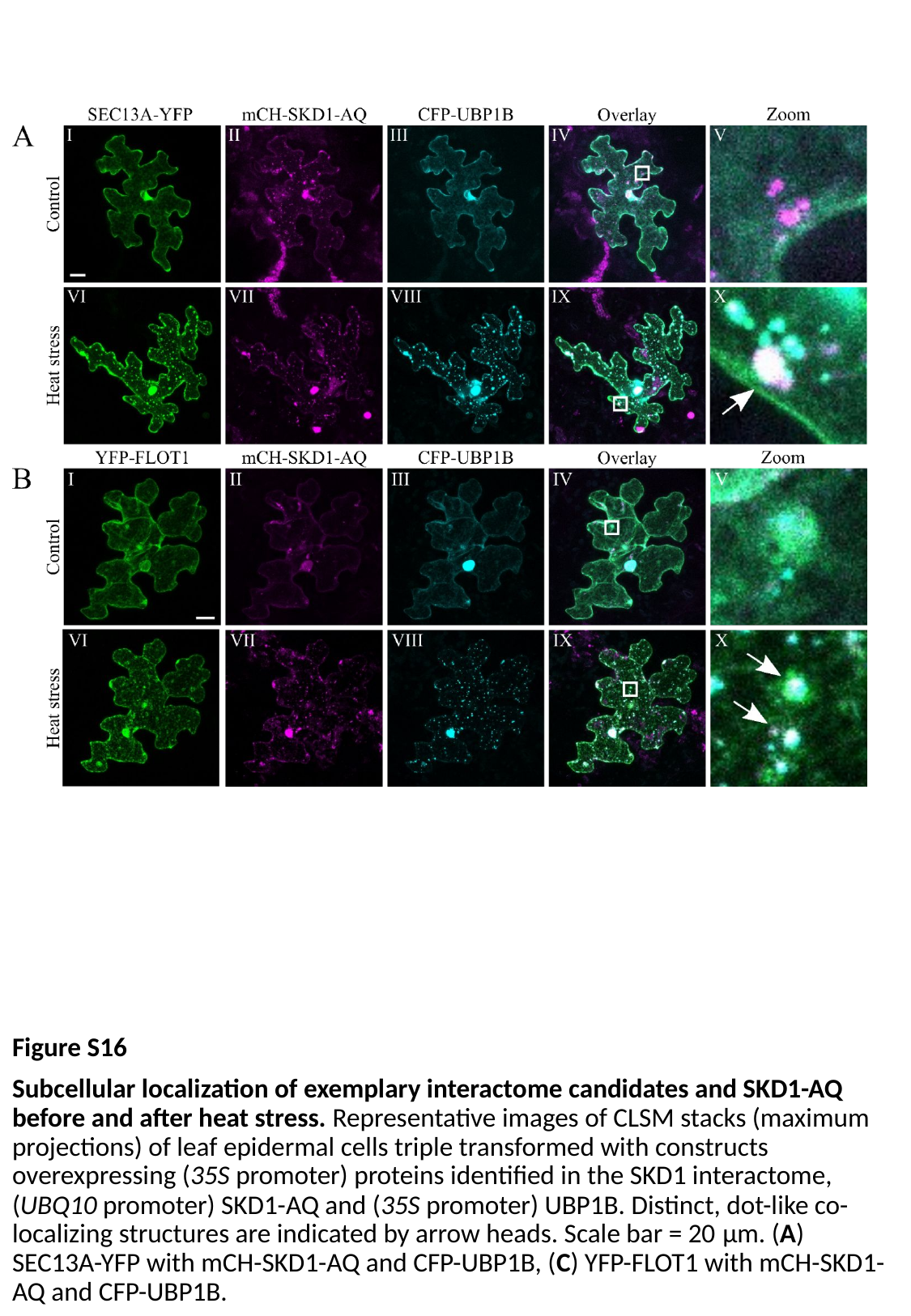

Figure S16
Subcellular localization of exemplary interactome candidates and SKD1-AQ before and after heat stress. Representative images of CLSM stacks (maximum projections) of leaf epidermal cells triple transformed with constructs overexpressing (35S promoter) proteins identified in the SKD1 interactome, (UBQ10 promoter) SKD1-AQ and (35S promoter) UBP1B. Distinct, dot-like co-localizing structures are indicated by arrow heads. Scale bar = 20 µm. (A) SEC13A-YFP with mCH-SKD1-AQ and CFP-UBP1B, (C) YFP-FLOT1 with mCH-SKD1-AQ and CFP-UBP1B.
